# Medication Samples and Smoking Cessation Among Adults: A Randomized Clinical Trial

**DOI:** 10.1001/jamanetworkopen.2026.11418

**Published:** 2026-05-08

**Authors:** Matthew J. Carpenter, Tracy T. Smith, Amy E. Wahlquist, Benjamin A. Toll, Karen L. Cropsey, Emily C. Ware, John M. Kaczmar, Jennifer Dahne, Kevin M. Gray

**Affiliations:** 1 Hollings Cancer Center, Medical University of South Carolina (MUSC), Charleston; 2 Department of Psychiatry & Behavioral Sciences, MUSC, Charleston, South Carolina; 3 Center for Rural Health and Research, East Tennessee State University, Johnson City; 4 Department of Public Health Sciences, MUSC, Charleston, South Carolina; 5 Department of Psychiatry, University of Alabama at Birmingham; 6 Department of Pharmacy, MUSC, Charleston, South Carolina; 7 Department of Medicine, MUSC, Charleston, South Carolina

## Abstract

**Importance:**

Smoking cessation interventions could ultimately offer greater impact to the extent that they are brief, concrete, and face valid (to the individuals presenting and receiving the intervention), which would then make these interventions scalable across a broad spectrum of adults who smoke (AWS). Medication sampling is one potential strategy to meet that need.

**Objective:**

To determine outcomes of varenicline sampling in a fully powered randomized clinical trial (RCT), conducted from February 2021 to April 2025.

**Design, Setting, and Participants:**

This decentralized RCT included non–treatment seeking AWS with varying levels of motivation to quit who were recruited throughout South Carolina.

**Intervention:**

Participants were randomized to receive a 4-week sample of varenicline, nicotine replacement therapy (NRT; active control), or quitline referral (inactive control) in a 2:1:1 ratio. Accompanying 4-week medication supply, intervention messaging in both sampling groups emphasized naturalistic use, ie, a participant-driven experience with self-determined uptake, use, and goals for use.

**Main Outcomes and Measures:**

The primary outcome was self-reported 7-day point prevalence abstinence (PPA) at 6-month follow-up. Secondary outcomes included carbon monoxide (CO)–verified abstinence, floating abstinence (any 7-day period of not smoking throughout follow-up), quit attempts, and smoking reduction.

**Results:**

The study sample included 651 AWS (mean [SD] age, 52 [11] years; 431 (66%) female), with 161 randomized to the no-sampling control, 172 randomized to NRT, and 318 randomized to varenicline. Compared with the no-sampling control, AWS receiving varenicline samples had higher rates of self-reported PPA at month 6 (16 of 161 [10%] vs 53 of 318 [17%]; *P* = .048), floating abstinence throughout follow-up (33 [20%] vs 108 [34%]; *P* = .003); and greater incidence of 50% or greater reduction in cigarettes per day (CPD) by 6 months (31 [19%] vs 106 [33%]; *P* = .002); however, rates of CO-verified abstinence at 6 months were not significantly different. Varenicline sampling was superior to NRT sampling for 6-month self-reported abstinence (53 [17%] vs 14 of 172 [8%]; *P* = .01); floating abstinence through 6 months (108 [34%] vs 43 [25%]; *P* = .04); and incidence of 50% or greater reduction in CPD at week 8 (111 [35%] vs 38 [22%]; *P* = .005) but not at 6 months.

**Conclusions and Relevance:**

In this RCT of 651 AWS, varenicline sampling was efficacious, with potentially superior outcomes compared with NRT. Results provide additional evidence in support of medication sampling as a pragmatic option to engage AWS in cessation, worth further evaluation within an applied settings.

**Trial Registration:**

ClinicalTrials.gov Identifier: NCT04525755

## Key Points

**Question:**

Does provision of varenicline samples, given broadly and with minimal guidance or instruction, facilitate smoking cessation among adults who smoke?

**Finding:**

This randomized clinical trial with 651 adults who smoke found that varenicline sampling led to increases in smoking cessation relative to no medication sampling and to marginal increases in cessation relative to sampling of nicotine replacement therapies.

**Meaning:**

Findings suggest that provision of a prescription cessation medication in an unguided context yields improvements in smoking cessation.

## Teaser

This randomized clinical trial evaluates whether offering adults who smoke samples of varenicline with minimal guidance or instructions increases smoking cessation rates.

## Introduction

Health care practitioners are continually challenged to provide effective smoking cessation interventions for the nearly 29 million adults who smoke (AWS) in the United States. This is particularly difficult within primary care, given the many demands on clinicians and the contracting resources (eg, time) to meet those needs. Many AWS remain unable or unwilling to quit,^1^ and for AWS making a quit attempt, utilization of evidence-based pharmacotherapies is very low: 9% to 16%.^2^ Novel approaches to cessation are needed, not only in terms of identifying new treatments but also in finding new ways to pragmatically apply them across any number of clinical settings. The latter might be particularly impactful given modeling research suggesting that increasing population penetrance of evidence-based quit methods will do more for population cessation rates than would increasing the efficacy of any given treatment.^3^ Our group has been testing the concept of medication sampling to meet that need. Medication sampling, originating with a focus on nicotine replacement therapy (NRT),^4,5^ is the provision of brief (2 to 4 weeks) cessation pharmacotherapy, administered to AWS who may or may not be motivated to quit, without any firm requirement or instruction to quit. It is an entirely user-driven experience applicable within almost any clinical context, including primary care,^6^ dental settings,^7,8^ and lung cancer screenings.^9^

Varenicline is a frontline cessation pharmacotherapy included in all recent clinical practice guidelines.^10,11^ Unlike NRT, varenicline is prescription-based only, involves titration of dosing, and has a moderately more pronounced adverse event profile, all of which could detract from the feasibility and/or efficacy of medication sampling. Building on our prior studies of NRT sampling, all generally favorable,^4,5,6^ we recently demonstrated that varenicline sampling is a feasible approach with potentially promising outcomes,^12^ worthy of a larger trial.

We herein report the primary outcomes of a decentralized randomized clinical trial (RCT) testing varenicline vs NRT vs no product sampling among a large sample of AWS with varying levels of motivation to quit. As the first large test of varenicline sampling of which we are aware, we focused on efficacy, not effectiveness. Thus, the study design was framed specifically around varenicline sampling, with comparisons with both an inactive (no product sampling) and active (NRT) sampling group.

## Methods

### Overview

AWS, with varying desire to quit smoking, were recruited across South Carolina and randomized into 1 of 3 groups: (1) varenicline sampling (4-week duration with self-determined titration of dosing), (2) NRT sampling (4-week duration of combination patch and lozenge; active control), or (3) quitline referral only (inactive control). Given the primary focus on varenicline sampling effects, participants were randomized in a 2:1:1 ratio. The primary outcome was smoking abstinence (7-day self-reported point prevalence abstinence) at 6-month follow-up. The trial was registered in advance and approved by the Medical University of South Carolina institutional review board (IRB), and all participants provided written informed consent. Recruitment began February 2021 and ended October 2024, with final data collection in April 2025. Reporting followed the Consolidated Standards of Reporting Trials (CONSORT) guidelines.^13^ The study protocol is provided within Supplement 1.

### Participant Eligibility, Screening, Consent, and Randomization

Recruitment advertising was posted on social media, with a direct link to an online screener to determine initial study eligibility, which required: (1) age 18 years or older; (2) residing in South Carolina; (3) daily smoking of at least 5 cigarettes on 25 or more days per month for at least 1 year; (4) not currently pregnant, breastfeeding, or planning to become pregnant; (5) no significant psychiatric contraindications (hallucinations, schizophrenia, bipolar disorder, suicidality) or seizures; and (6) ownership of smartphone and/or regular access or use of email or text messaging. In addition, initial study criteria required that each participant have a primary care clinician, under the rationale that continued use of varenicline would require a health care practitioner to prescribe it: we did not want to provide varenicline if there was no possibility of it being continued after the sampling period. However, upon study onset, this criterion quickly became the leading cause of screening failure (834 screen failures within 4 months), which compromised long-term sample recruitment. We thus removed this criterion, and for the remainder of the study offered referrals to our university-affiliated Tobacco Treatment Program for anyone needing assistance with medication continuation.

Upon initial screening, potential participants were scheduled for virtual consent and secondary screening with study staff, which consisted of informed consent procedures conducted online via a secure platform for consent sharing, discussion, and signing of consent. After consent, staff again screened for acute psychiatric conditions, including suicidality, with IRB-approved precautions to manage any emergent concerns. Women aged 55 years and younger were mailed a pregnancy test kit with instructions to take and subsequently access an online form attesting to a negative result. Upon clearance of these precautions, the study physician conducted a clinical review to determine study eligibility.

At the week-0 phone call, study staff collected a baseline questionnaire consisting of demographic characteristics and smoking history. Participants self-reported their race as Black or African American, multiracial, White, or additional groups (American Indian or Alaska Native, Asian, and Native Hawaiian or Other Pacific Islander individuals as well as those who self-reported Other race). Participants were then randomized via mixed block design (nQuery) to varenicline vs NRT vs no sampling treatment conditions (2:1:1) utilizing block sizes in multiples of 4 (ie, block sizes of 4, 8, 12), stratifying on motivation to quit (low, <7; or high, ≥7; on 0-10 scale^14^). Study staff engaged participants in a brief description of each group as discussed below. There was no other direct engagement with study participants beyond week 0. At the conclusion of the week-0 phone call, all participants were mailed a device for remote collection of carbon monoxide (CO) to verify claims of smoking abstinence.

### Sampling Interventions

Participants in the varenicline group were mailed 1 bottle of 0.5-mg tablets (56 count), with general guidance that they could use it as they wish. Full intervention messaging, both oral (provided at baseline call) and written (provided within mailing), is provided in eAppendix 1 in Supplement 2. Dosing recommendations, described in full within the mailed brochure, were standard, as per clinical practice: 0.5 mg/d, titrated up to 2 mg/d if so desired. Thus, the sampling experience was intended to last up to 4 weeks if taking 1 mg/d (2 pills for 28 days) but could be shorter if participants titrated up to 2 mg/d (4 pills for 14 days). In line with our prior sampling studies, a 4-week period was chosen as a reasonable duration to experiment and acclimate to the medication. Study staff clarified any questions and discussed most common adverse effects (insomnia, disturbed sleep, nausea) and ways to minimize them, which was also included within mailed handout. Throughout, the emphasis was on participant-driven sampling experience with self-determined uptake, utilization, and goals for using varenicline.

Participants in the NRT group were mailed a 4-week supply of nicotine patches (14 mg) and lozenges (4 mg) with comparable instructions for self-determined use. Dosing was standardized to enhance the downstream potential to deliver NRT sampling within clinical settings, where tailoring may not be feasible. NRT messaging, both oral and written, is provided in eAppendix 1 in Supplement 2.

Participants in the control group were not provided any medication samples but were told that they could use cessation medication if they so chose, on their own. All participants across all groups were given standard cessation support materials and specific contact information and benefits of using the SC Quitline.

### Assessments

Follow-up surveys were sent at weeks 2, 4, 8, 12, and 24, all via text or email with links to online assessments. Smoking-related outcomes included: (1) self-reported 7-day point prevalence abstinence from smoking (primary); (2) CO-verified 7-day point prevalence abstinence (≤6 parts per million), assessed via iCO Smokerlyzer mailed to all participants; (3) the cumulative incidence of having ever achieved 7-day not-smoking (self-reported) throughout follow-up (ie, floating abstinence); (4) the cumulative incidence of any 24-hour quit attempt throughout follow-up; and (5) reduction in smoking, defined as the proportion of participants within each group who reduced cigarettes per day (CPD) by 50% or more since baseline. Medication uptake was defined as use of medication (yes or no) and regular use of medication (≥4 d/wk).

Potential mechanisms of medication sampling included: (1) motivation and confidence to quit (both assessed on 0-10 visual analog scales) and (2) cessation fatigue,^15^ assessed via 8 items with Likert responses scored 1 to 5 that tapped into emotional exhaustion from quitting (averaged; possible range 1-5; higher scores indicative of greater exhaustion). Attention checks (eg, “enter 5 here”) were periodically embedded throughout online survey assessments. Repeated failures across these items within a survey resulted in removal of a time point (≤2% within any given time point) or, if repeated over multiple surveys, complete removal of the participants from the intent-to-treat (ITT) sample.

### Statistical Analysis

A priori power estimations were based on the primary outcome: 7-day point-prevalence abstinence at 6 months, focused on the primary comparison of varenicline vs inactive (no-sampling) control. Based on our early work, we anticipated an abstinence rate of 18% in the varenicline group vs 8% in the control group, the latter also in line with our prior studies.^6^ With 2:1 randomization for this comparison and 80% power, this difference required a total sample size of 413 participants across these 2 groups. We inflated this by 15% to account for potential attrition, resulting in 324 varenicline vs 162 control planned participants. With an added comparator group of NRT sampling (n = 162; 2:1:1 overall randomization), we anticipated a total sample size of 648 participants.

Outcomes for both NRT and control groups are presented in the Results section, but analyses did not include formal comparison between them since (1) the primary focus was on varenicline sampling and (2) the study was underpowered for this comparison. Descriptive statistics, including frequency, percentages, means, and SDs, as appropriate, were calculated to describe the study sample, both overall and by group. Binary outcomes, including all abstinence-related outcomes, were primarily analyzed via generalized linear mixed models (GLMMs) to examine outcomes between groups over time. GLMMs utilized logit links and random residual options to inform time points with missing data with data collected at earlier assessments, within an individual. Models included main effects of group (varenicline vs NRT vs control) and time (weekly assessments at weeks 2, 4, 8, 12, and 24) and their interaction. Covariate adjustment (lifetime history of psychiatric comorbidity) was included as well given baseline group differences (per protocol). eAppendix 2 in Supplement 2 presents alternative modeling options, including unadjusted outcomes. For interaction terms that were not statistically significant (*P* > .05), GLMMs were rerun after removing the interaction term to interpret the main effects of group and time directly. For specific time point comparisons to examine pairwise group differences, logistic regression models were utilized with contrast statements to compare group combinations. In these logistic regression models, individuals with missing data for specific time points were treated as not having the outcome of interest only at that specific time point. No other imputations were made. Results from these models are reported as odds ratios (ORs) with 95% CIs. Continuous outcomes used a general linear model framework to examine changes over time, similar to the binary outcomes utilizing GLMMs. For continuous outcome analyses, the week-0 value for each outcome was included as a covariate along with history of psychiatric comorbidity, main effects of group and time, and their interaction. As in other analyses, when the interaction term was not statistically significant, it was removed from the model to directly interpret the main effect of group. For both continuous outcomes and medication use analyses, no imputation was done; missing data remained missing. We used χ^2^ tests to compare medication use between varenicline and NRT groups only, as medication use in the control group was expected to be low. Finally, in line with our prior sampling studies wherein we conducted stratified analyses among participants with low (scores 0-6) vs high (scores 7-10) motivation to quit, we did the same here for exploratory purposes only, using methods described previously. Statistical analyses were conducted with SAS version 9.4(c) (SAS Institute), and *P* < .05 was considered statistically significant.

## Results

**Figure 1.**
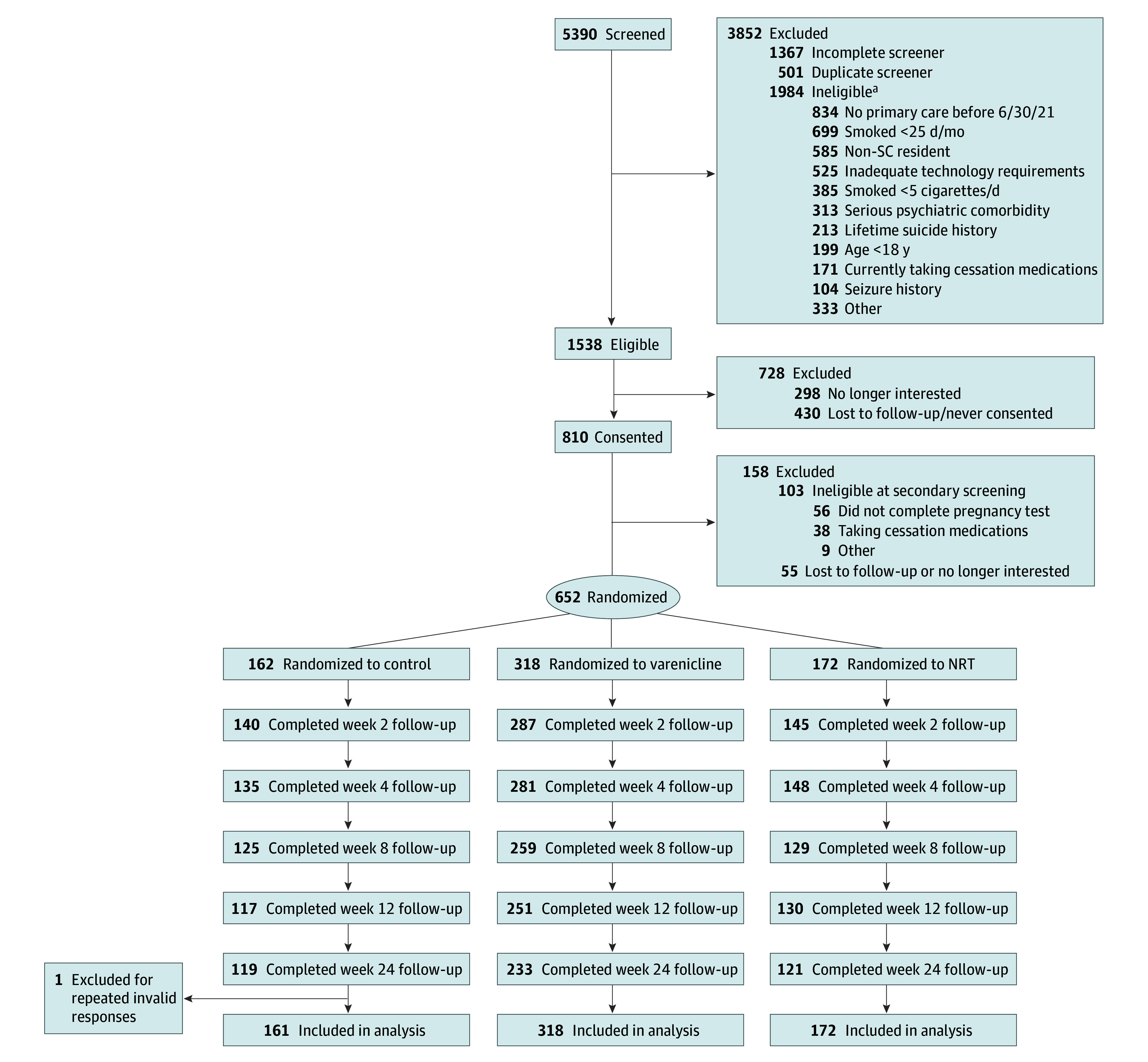
Study Flowchart NRT indicates nicotine replacement therapy; SC, South Carolina. ^a^Not mutually exclusive.

Of 651 enrolled participants, the mean (SD) age was 52 (11) years; there were 431 female participants (66%); 74 Black (11%), 11 (2%) multiracial, and 555 (85%) White participants; and 460 (71%) had at least some college-level educational attainment (Table). A total of 161 were randomized to the nonsampling control group, 172 to NRT, and 318 to varenicline. There were no differences between groups on any baseline variable except lifetime psychiatric comorbidity, highest within the NRT group (60 [35%]) and lowest within the varenicline group (78 [25%]). Thus, all analyses for cessation-related outcomes control for lifetime psychiatric comorbidity. As seen in Figure 1, there were no differences in study retention across groups.

**Table.** Baseline Participant Characteristics

Characteristic	Participants, No. (%)			
	Overall (N = 651)	Control (n = 161)	NRT (n = 172)	Varenicline (n = 318)
Age, mean (SD), y	51.9 (11.4)	51.5 (11.5)	52.0 (10.4)	52.1 (11.9)
Gender				
Female	431 (66.2)	102 (63.4)	119 (69.2)	210 (66.0)
Male	219 (33.6)	59 (36.6)	52 (30.2)	108 (34.0)
Race				
Black or African American	74 (11.4)	15 (9.3)	23 (13.4)	36 (11.3)
Multiracial	11 (1.7)	4 (2.5)	2 (1.2)	5 (1.6)
White	555 (85.3)	137 (85.1)	147 (85.5)	271 (85.2)
Additional groups^a^	11 (1.7)	5 (3.1)	0 (0.0)	6 (1.9)
Highest education				
HS, GED, or partial HS	191 (29.3)	47 (29.2)	52 (30.2)	92 (28.9)
College, 1-3 y	327 (50.2)	81 (50.3)	88 (51.2)	158 (49.7)
College, ≥4 y	133 (20.4)	33 (20.5)	32 (18.6)	68 (21.4)
Household income, %				
<25 000	179 (27.5)	40 (24.8)	50 (29.1)	89 (28.0)
25 000 to <50 000	218 (33.5)	56 (34.8)	66 (38.4)	96 (30.2)
50 000 to <75 000	111 (17.1)	34 (21.1)	22 (12.8)	55 (17.3)
≥75 000	130 (20.0)	28 (17.4)	33 (19.2)	69 (21.7)
Not currently insured	158 (24.3)	40 (24.8)	43 (25.0)	75 (23.6)
Unemployed	96 (14.7)	29 (18.0)	26 (15.1)	41 (12.9)
Lifetime psychiatric comorbidity^b^				
Depression	133 (20.4)	39 (24.2)	42 (24.4)	52 (16.4)
Anxiety	112 (17.2)	26 (16.1)	31 (18.0)	55 (17.3)
Other mental health disorder	14 (2.1)	3 (1.8)	7 (4.1)	4 (1.3)
Alcohol use disorder	15 (2.3)	3 (1.9)	4 (2.3)	8 (2.5)
Other substance use disorder	10 (1.5)	3 (1.9)	3 (1.7)	4 (1.3)
Any lifetime psychiatric disorder^c^	190 (29.2)	52 (32.3)	60 (34.9)	78 (24.5)
Smoking characteristics				
Cigarettes per day, mean (SD), No.	18.3 (8.5)	18.3 (8.2)	18.3 (8.7)	18.4 (8.6)
Age began smoking, mean (SD), y	17.8 (5.9)	17.9 (6.0)	17.2 (5.4)	18.0 (6.1)
Past year quit attempt	192 (29.5)	49 (30.4)	51 (29.7)	92 (28.9)
Motivation to quit, mean (SD)^d^	8.1 (2.2)	8.0 (2.1)	8.0 (2.3)	8.1 (2.2)
Confidence to quit, mean (SD)^d^	6.3 (2.4)	6.2 (2.4)	6.3 (2.5)	6.3 (2.3)
Prior use of varenicline	182 (28.0)	47 (29.2)	37 (21.5)	98 (30.8)
Prior use of NRT	451 (69.3)	110 (68.3)	124 (72.1)	217 (68.2)
Live with other AWS in home	233 (35.8)	52 (32.3)	55 (32.0)	126 (39.6)

Abbreviations: AWS, adult who smoke; GED, General Educational Development; HS, high school; NRT, nicotine replacement therapy.

^a^ Additional groups included American Indian or Alaska Native, Asian, and Native Hawaiian or Other Pacific Islander individuals as well as those who self-reported Other race.

^b^ Comorbidities assessed with the question, “Have you ever been told by a doctor or other health professional that you had any of the following?”

^c^ Any response of yes to the list of comorbidities.

^d^ Measured on a 0 to 10 scale, with higher scores indicating greater motivation or confidence.

### Abstinence-Related Outcomes: Self-Reported, CO-Verified, and Floating Abstinence; Quit Attempts; and Smoking Reduction

**Figure 2.**
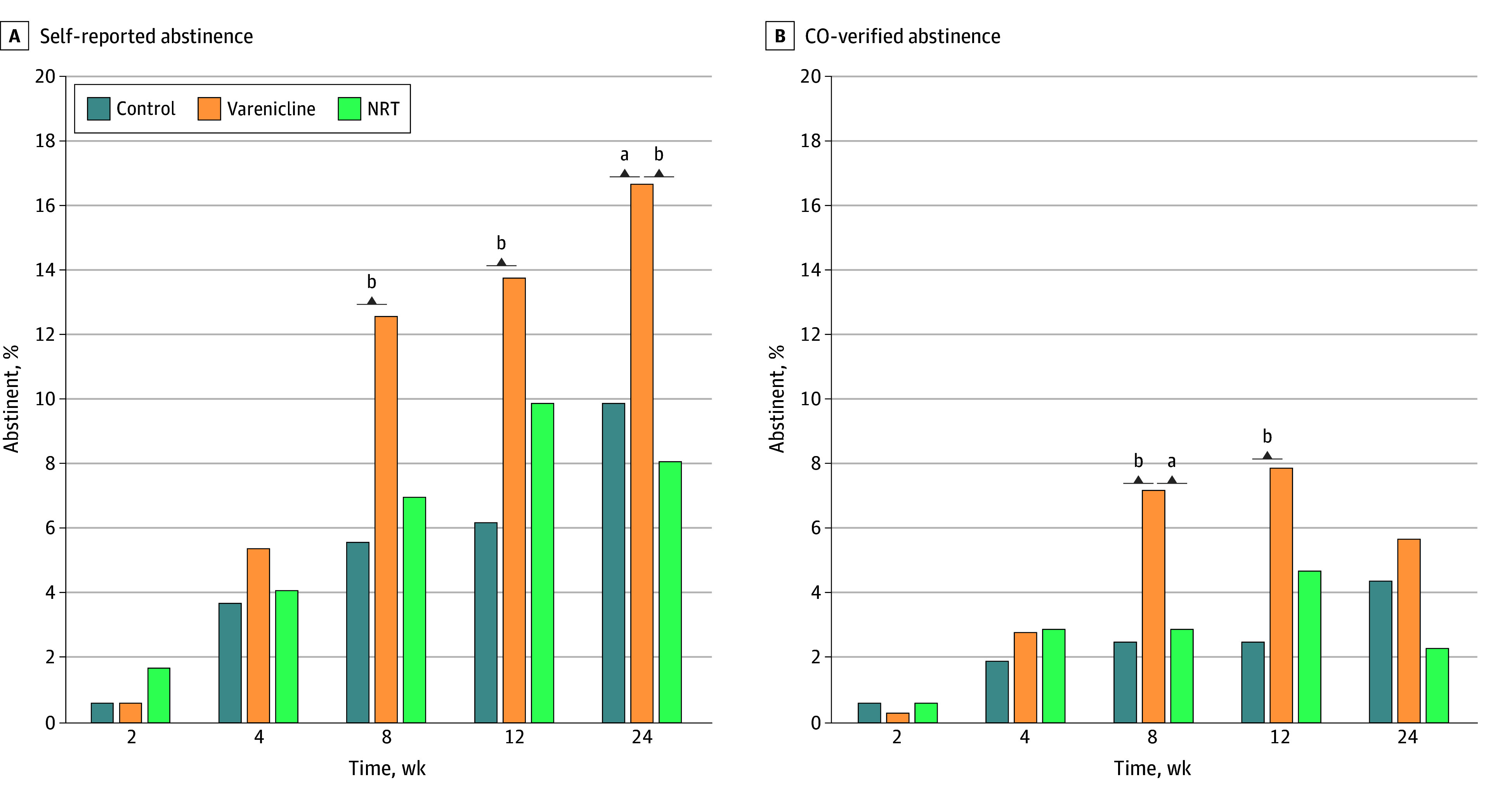
Bar Graphs of Self-Reported and Carbon Monoxide (CO)–Verified Abstinence Figure presents self-reported 7-day point prevalence abstinence (A) and CO-verified 7-day point prevalence interaction (B). Percentages based on raw percentages from available data, not generalized linear mixed model estimates. Neither time × group interaction was statistically significant (self-reported: *P *for interaction = .49; CO-verified: *P *for interaction = .37). NRT indicates nicotine replacement therapy. ^a^*P* < .05. ^b^*P* = .05.

For self-reported abstinence, the overall group × time interaction was not significant (*P *for interaction = .49). However, there was a significant effect for treatment group (*P *for interaction = .02) such that those in the varenicline group achieved and sustained significantly higher rates of self-reported smoking cessation compared with those in both the control (OR, 2.16; 95% CI, 1.14-4.08) and NRT (OR, 2.03; 95% CI, 1.10-3.77) groups (Figure 2). Pairwise comparisons between varenicline and control were statistically significant at weeks 8 (44 of 318 [13%] vs 10 of 161 [6%]; *P* = .02), 12 (40 [14%] vs 9 [6%]; *P* = .02), and 24 (53 [17%] vs 16 [10%]; *P* = .048), while a pairwise difference between varenicline and NRT was found at week 24 only (53 [17%] vs 14 of 172 [8%]; *P* = .01).

Across all groups and across all follow-up time points, there were 251 instances of self-reported abstinence, of which 131 (52%) included a returned CO sample (eAppendix 3 in Supplement 2). Neither the group × time interaction (*P *for interaction = .37) nor the main effect of group, after removing the interaction term (control, 4%; varenicline, 8%; NRT, 5%; *P* = .12), retained statistical significance at the a priori level. Nonetheless, pairwise differences in CO-verified abstinence between varenicline and control were found at weeks 8 (23 [7%] vs 4 [2%]; *P* = .04) and 12 (25 [8%] vs 4 [2%]; *P* = .03), while no pairwise differences were found between varenicline and NRT, with the exception of week 8 (23 [7%] vs 5 [3%]; *P* = .046).

For rates of floating abstinence (ever achieving 7 days of not smoking throughout follow-up), there was no statistically significant group by time interaction (*P *for interaction = .91), but there was a significant main effect for group (*P *for interaction = .01) such that participants in the varenicline group achieved higher ever-quit rates throughout follow-up compared with participants in the inactive control group (108 [34%] vs 33 [20%]; *P* = .003; OR, 2.72; 95% CI, 1.39-5.35). Participants in the varenicline group had higher floating abstinence rates than those in the NRT group through 6 months (108 [34%] vs 43 [25%]; *P* = .04).

**Figure 3.**
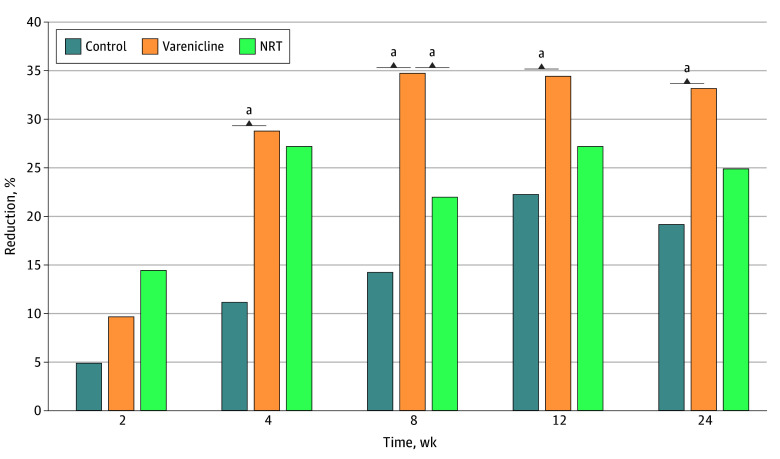
Bar Graph of Smoking Reduction Over Time Figure presents incidence of a 50% or greater reduction in cigarettes per day from week 0, averaged over preceding 7 days at each follow-up time point. Smoking reduction interaction term (time × group) was statistically significant (*P *for interaction = .006). NRT indicates nicotine replacement therapy. ^a^*P* < .05.

For the incidence of 24-hour quit attempts, assessed serially and cumulatively throughout follow-up, neither the group × time interaction (*P *for interaction = .67) nor the main effect of group (control, 16%; varenicline, 24%; NRT, 19%; *P *for interaction = .24) was statistically significant. However, there was a significant group × time interaction for smoking reduction, assessed as the incidence of 50% or greater reductions in CPD over time (*P *for interaction = .006) (Figure 3). There was greater incidence of this reduction in CPD in participants in the varenicline group compared with the inactive control group at 6 months (106 [33%] vs 31 [19%]; *P* = .002) as well as compared with the active control group at week 8 (111 [35%] vs 38 [22%]; *P* = .005).

### Mechanisms: Medication Uptake, Motivation and Confidence to Quit, and Cessation Fatigue

**Figure 4.**
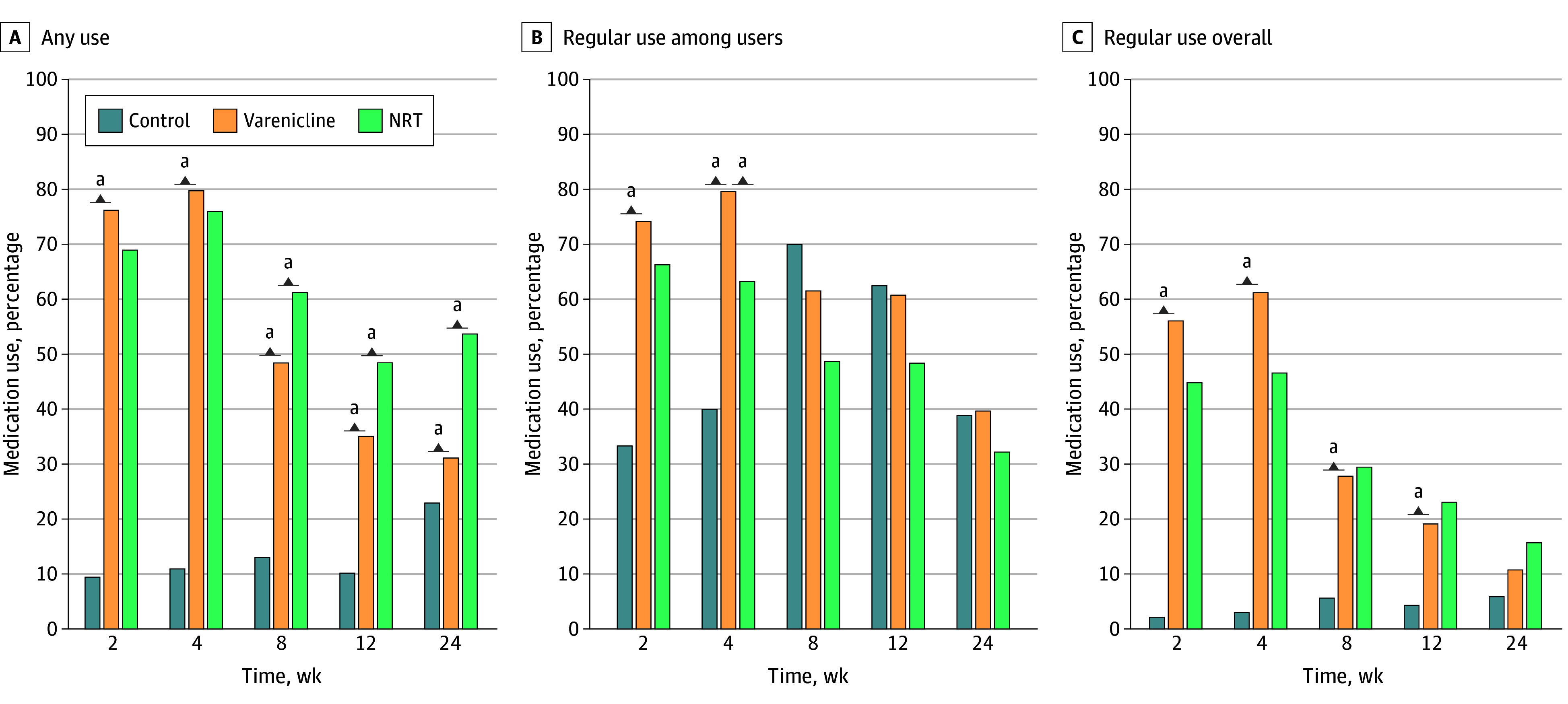
Bar Groups of Medication Use of Varenicline and/or Nicotine Replacement Therapy (NRT) A and C, Results based on respondents only (not intention-to-treat population). B, Results based on medication users only, at each time point. Regular use was defined as using either varenicline or NRT (patch or lozenge) for 4 or more days per week. Statistical comparisons were conducted only between varenicline vs NRT and varenicline vs control. Individuals in any of the 3 treatment groups could have reported use of any medication. ^a^*P* < .05.

Rates of using sampling medications (varenicline and NRT, specifically, patch and/or lozenge) are shown in Figure 4. To allow for statistical comparisons between groups, Figure 4 shows rates of using either varenicline or NRT. As expected, use of medication was significantly higher within the varenicline group than the control group throughout follow-up. Rates of any use were generally higher within the NRT group compared with the varenicline group but only after the sampling period was over (weeks 8-24). There were no differences between varenicline and NRT groups in regular use (defined as using ≥4 days per week, asked only among medication users).

The group × time interaction was statistically significant for both confidence to quit (*P *for interaction < .001) and cessation fatigue (*P *for interaction = .001) but not motivation to quit (*P* = .05). Motivation and confidence to quit declined in the control group (particularly over first 4 weeks of follow-up) whereas in both medication sampling groups, the outcomes initially held steady, followed by a decline later in the follow-up period (eAppendix 4 in Supplement 2).

### Exploratory Outcomes

In our previous similarly designed studies of NRT sampling, we have reported outcomes within subgroups of AWS who expressed low vs high motivation to quit at baseline, under the rationale that sampling might be as or more beneficial to those AWS who lack motivation to quit and who may benefit from a behavioral nudge. As with these prior studies, raw rates of all cessation-related outcomes in the current trial were higher among AWS with strong quit motivations. However, the directionality and magnitude of effect sizes are generally comparable among AWS for both low and high quit motivation. eAppendix 5 in Supplement 2 presents all results stratified by baseline motivation to quit. Finally, there were no IRB-defined serious adverse events throughout the study, a more detailed profile of which is provided within eAppendix 6 in Supplement 2.

## Discussion

Trial results demonstrate the beneficial impact of varenicline sampling, provided with minimal instruction across a broad range of AWS. These benefits were seen across a number of cessation-related outcomes, providing robust confidence in trial interpretation. These results are consistent with other studies of varenicline as delivered over-the-counter (OTC),^16^ mailed,^17,18,19^ among unmotivated AWS explicitly,^20,21^ and/or in the absence of a formal clinical interview.^18,19^ It is clear that provision of prescription cessation medication, even if typically prescribed, is feasible and effective in an unguided, sampling context. While this study was not conducted within a health care setting (consistent with focus on efficacy over effectiveness), this seems the logical next step, ie, to determine whether varenicline sampling can be disseminated as a pragmatic treatment option within a clinical context.

This trial was neither focused nor powered on evaluation of NRT sampling, which was included merely as an active control group and to offer preliminary comparisons with varenicline. Nonetheless, a few observations are possible. Absolute differences in NRT vs control outcomes were not remarkably discrepant across studies,^4,5,6^ suggesting that a larger sample size would yield similar statistical significance for these comparisons. What is clear from the current trial is that varenicline led to numerically, if not statistically, higher cessation outcomes compared with NRT, issues of power notwithstanding.

As with our prior study of NRT sampling within primary care,^6^ varenicline sampling led to generally comparable treatment effects (though not raw outcomes) across AWS with low and high motivation to quit. We did not formally test for a treatment interaction, and we were not powered to do so. Nonetheless, these and prior results are consistent with the notion of behavioral nudges,^22,23^ aimed at changing behavior through positive structuring of choice architecture,^24^ ie, preserving individual autonomy to self-direct health behavior change.

### Limitations

The trial was not without limitations, predominantly the poor adherence to remote CO-verification of abstinence. The study was conducted at a time, post–COVID-19, when procedures for remote biomarker capture were in their early evolution, guidance for which has since been published elsewhere.^25,26^ Additionally, CO verification could be confounded by smoked cannabis use, which was unassessed. Underrepresentation of male AWS, those with low motivation to quit, and those from minoritized racial and ethnic groups represents other study limitations, potentially limiting generalizability. Additionally, the study was restricted to South Carolina (complying with state-level medical and pharmacy licensing), perhaps also limiting generalizability.

## Conclusions

This RCT found that varenicline sampling was efficacious, with outcomes at least as efficacious as, and potentially superior to, NRT. This trial provided additional evidence in support of medication sampling as a pragmatic option to engage AWS in the cessation process, worth additional evaluation in a health care setting.

## Notes

Supplement 1.
Trial Protocol

Supplement 2. **eAppendix 1.** Treatment Materials Within Medication Sampling Groups **eAppendix 2.** Alternative Models, With and Without Adjustment **eAppendix 3.** CO Adherence With Self-Reported Abstinence, per Group, per Time Point **eAppendix 4.** Treatment Mechanisms **eAppendix 5.** Analyses by Motivation to Quit **eAppendix 6.** Adverse Events

Supplement 3.
Data Sharing Statement
