# Supplementary material for: Medication Samples and Smoking Cessation Among Adults: A Randomized Clinical Trial
Source: JAMA Netw Open. 2026 May 8;9(5):e2611418. doi: 10.1001/jamanetworkopen.2026.11418 (PMC13156786; doi:10.1001/jamanetworkopen.2026.11418)
Supplement: Supplement 1. — Trial Protocol [file jamanetwopen-e2611418-s001.pdf]

Grant Title: A Translational Randomized Clinical Trial of Varenicline Sampling to Promote Smoking Cessation and Scalable Treatment Dissemination

Short Title: STARS (Smoking Treatment And Remote Sampling) Study

Principal Investigator: Matthew Carpenter, PhD

Funding Source: NCI (R01 CA246729)

Registration: NCT 04525755

Original Approval Date: 3/11/2020

Last Approval Date (with protocol change): 10/14/2024

Date of Final Amendment (without protocol change): 5/28/2025

I. Background & Significance

Despite advances in treatment of tobacco dependence, smoking continues to be the leading cause of preventable mortality, resulting in an estimated 480,000 U.S. deaths annually(1) and incurring nearly $170 billion in direct medical care for adults.(2) Tobacco control is cancer control, and novel approaches to cessation are needed. Such efforts must not only identify new treatments, but also find new ways to apply them at the population level. Given the lengthy duration to identify and test new treatments, we focus on the latter: wider implementation of existing treatments. Public health efforts must increase the incidence of evidence-based quit attempts (i.e., use of FDA-approved cessation pharmacotherapies), a clear goal of Healthy People 2020 recommendations. Indeed, modeling research suggests that increasing population penetrance of evidence-based quit methods will do more for population cessation rates than would increasing the efficacy of any given treatment.(3) USPHS clinical practice guidelines(4) and other meta-analyses(5-8) have made clear recommendations that 1) all tobacco users should be offered brief advice to assist in quitting, and 2) unless medically contraindicated, all smokers should be offered pharmacotherapy. However, our studies(9, 10) and those of many others(11-18) consistently demonstrate that use of pharmacotherapies is unacceptably low, with <25% of quit-attempters using these treatments (See Figure 1; not counting the many smokers not making a quit attempt).

Figure 1: Treatment Utilization Among Smokers Making a Quit Attempt in Past Year (Source: TUS-CPS)

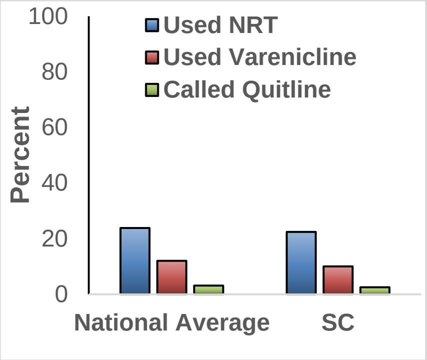

Individual Barriers to Evidence Based Quit Attempts

Nicotine dependence is the primary cause of persistent smoking at the individual level.(19) But there are additional reasons as to why many smokers are unable or unwilling to engage in an evidence-based quit attempt. Many smokers make ill-supported, i.e., unassisted quit attempts over time,(20) creating a “rollercoaster” of motivation, confidence, and willingness to try again.(21) Our research group has recently explored the notion of “cessation fatigue,” i.e., general feelings of burnout, hopelessness, and pessimism, that likely drives and derives from this rollercoaster. Building on our formative work to develop a multidimensional scale of cessation fatigue,(22) we subsequently demonstrated that cessation fatigue prospectively predicts important cessation milestones (both initiation of quit attempt and lapse/relapse) and thus is a novel target for cessation intervention(23) Elsewhere, we(24, 25) and others(12, 26-28) have also shown that many smokers are unfamiliar with cessation medications, or hold misperceptions about them that likely undermine their use. These views are particularly entrenched among smokers of lower socioeconomic status,(25, 29, 30) which further undermines pharmacotherapy use among these groups, yet again undermining treatment engagement at the population level. Collectively, these factors work to undermine a smoker’s self-control over the quitting process, giving them fewer tools to succeed. Much like a muscle that needs to be exercised,(31, 32) self-control can also be strengthened, which has been empirically demonstrated to improve tobacco abstinence.(33) There is thus an important public health need to capitalize on existing treatments and deliver them in ways that specifically enhance a smoker’s capacity to initiate and sustain evidence-based quit attempts. Such methods should bolster smoker motivation and confidence, increase familiarity with and acceptance of treatments, and enhance self-control, all in an effort to increase the incidence and success of quit attempts at the population level. Such interventions should be brief, concrete, and offer potential for scalability into larger populations of smokers. We believe that medication sampling meets this need (Figure 2: Conceptual Model). Whether and how these mechanisms differ for varenicline vs. NRT sampling is unclear. Though this is not the central focus on this application, we will be able to test this.

Figure 2: Conceptual Model to Increase Evidence Based Quit Attempts

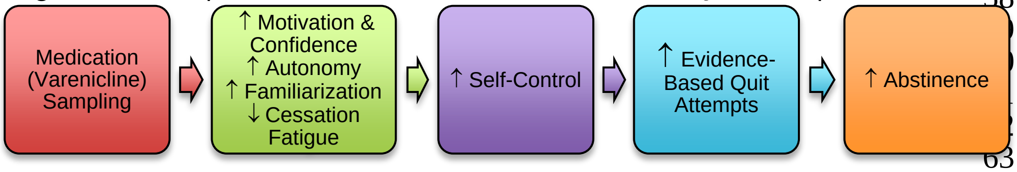

__Medication Sampling__

Medication sampling simply refers to providing starter packs of cessation medications. Unlike traditional models that first rely on smoker intent to quit, sampling is readily scalable to smokers regardless of their quit intention, offering a concrete, behavioral cue to facilitate quitting. It is not meant to replace more intensive treatment, but rather serve as a catalyst to it. We would never suggest that sampling will yield similar cessation outcomes compared to full-course treatment (it will not; see Table 1 below). Sampling simply extends reach

and gives smokers an immediately actionable evidence-based treatment method, designed primarily to heighten smoker acceptance and enhance motivation and self-confidence.

Table 1 demonstrates the population impact of current strategies for smoking cessation, assuming that 55% of smokers make a quit attempt,(34) 25% of them use cessation medication,(9) and 25% these will result in quitting success.(4) Medication sampling could increase population impact even if it resulted in a nominal increase in incidence of quit attempts, if not all quit attempters use the medication (a low estimate, given universal, free provision of product) and even if quit success is *markedly lowered* as compared to traditional RCT outcomes (as we would expect). Each of the above inputs for medication sampling are supported by our preliminary data, below. These data clearly show the potential of medication sampling at the population level.

Table 1: Potential Population Impact of Medication Sampling

	Current Evidence	Medication Sampling
Probability (P) of Quit Attempt (x)	.55	.65
(P) of Using Evidence-Based Quit Method (y)	.25	.8
(P) Success Per Method (z)	.25	.1
Impact: Population Quit Rate (x*y*z)	3.4%	5.2%

Uptake of cessation treatment is higher when medication accessibility is increased.(35) One simple but very interesting study assessed stages of change among two separate groups of smokers.(36) One group was asked about their intentions to quit, and the other group was asked the same question, but was preceded by a free offer of a cessation medication. The medication “carrot” significantly altered the distribution of motivation, with more smokers expressing quit interest. Translating this to cessation outcomes is more important, and is evidenced by our preliminary data, below. Many quitline studies show that provision of free medication improves cessation rates.(37-42) Some(38, 43) but not all(39) research even suggests that 2-week provision of NRT is as cost-effective for promotion of quitting as is extended supply.

Our view of sampling is supported by the quitline literature above but extends beyond it in both conceptualization and methods. Quitlines reach a very small portion (~3%) of the smoker population.(9) They also reach a very narrow population: smokers who want to quit. And they provide medications in very structured ways, with guided instructions for cessation-focused use, and with support calls to follow. In contrast, our view of sampling is more naturalistic and self-determined. We target a wider group of smokers, including those not wanting to quit, with the belief that sampling is a concrete, behavioral catalyst (cue to action), consistent with major theories of health behavior change.(44-46) Instructions are kept minimal to increase translational, scalable potential for eventual provision within clinical settings. While not an explicit outgrowth of self-determination theory,(47, 48) medication sampling is wholly consistent with it; i.e., with the rationale that smokers will be more invested in the quitting process if they decide for themselves the goals and pace for changing tobacco use.

Varenicline Sampling: Similar Benefits (or Not)?

Varenicline is a well-established cessation medication(49-53) that reduces withdrawal and blunts the reinforcing effects of nicotine.(54) Its efficacy is well-documented, known to be superior to all other monotherapies.(49, 50, 55-57) One survey of >900 medication users assessed a range of satisfaction measures across a range of medications.(58) Varenicline yielded the strongest endorsement of any: 77% called it an “excellent” medication (vs. 67%, the highest for any NRT product); 37% said it reduced tobacco consumption prior to a quit attempt (vs. 19%, the highest for any NRT). Among smokers who used *both* varenicline and NRT (a *within participant* comparison), most favored the former over the latter, and 77% would suggest varenicline to a friend (vs. 59% for patch users, the highest of any NRT). These data show smoker preference for varenicline despite: a) prescription status, b) higher side effect profile, and c) anecdotal reports of medication harm. Prior to 2015, we could never have proposed a hands-off sampling study of varenicline, due to FDA black box warnings. The EAGLES trial(56, 57) changed that, as did a number of other persuasive reviews,(50, 59-62) all of which demonstrated a strong safety profile, even among smokers with psychiatric conditions. The black box warnings were removed, which now allows for greater population dissemination. Given prior studies that examined flexible administration of varenicline, including reduce-to-quit,(63, 64) flexible quit date(65) and lower dosing protocols,(66, 67) a sampling approach offers another compelling option to increase use and accessibility of our strongest medication option.

Prior studies of short-term varenicline delivery; Prior studies of novel varenicline delivery

We could find no studies that conform to our approach per se, but other studies offer indirect support. Two quitline studies examined the effects of varenicline distribution. The first was a non-randomized comparison between smokers who self-selected to receive varenicline or NRT via their quitline.(68) Abstinence at 3 months (RR=1.7) and 6 months (RR=1.5) favored varenicline. Three design elements constrain interpretation. First,

these groups were non-randomized, and it was clear that varenicline users were very different from NRT users (older, with longer duration of smoking). Second, there was no biological verification of smoking. Third, there was no non-sampling comparison. These are all issues we address herein. The other quitline study is a recent trial from Dr. Toll (co-investigator) that randomized smokers to receive mailed NRT (n=100) or vouchers for varenicline (n=200), to be redeemed for free at a local pharmacy.(69) Only 27% of smokers in the varenicline group redeemed the voucher, which limited tests of abstinence. This suggests that free coverage of medications alone is not sufficient, and that there needs to be more immediate access: i.e., actually giving it to the smoker. Again, the study did not include a non-sampling control group, nor did it biologically verify smoking outcomes.

Outside the quitline context, and beyond short-term delivery, two studies examined varenicline as recommended (12 weeks), but with nuanced delivery that is highly relevant here. One was a study from Ontario that examined full course (12 weeks) varenicline vs. bupropion.(70) This was NOT a sampling study, adjunctive support was not minimal (extensive motivational emails throughout follow-up), nor was it open to a wide spectrum of smokers (eligibility based on wanting to quit in next 30 days). It is presented here because direct physician involvement was non-existent. Eligibility, assessment, and provision of prescription were managed entirely through an electronic process. Abstinence outcomes significantly favored the groups that received varenicline (RR=1.9) but not bupropion (RR=1.5), relative to smokers who did not receive medication. Like the Toll study above, uptake of medications through faxed prescription to a local pharmacy resulted in modest initiation and use of the medication, which again suggests that physical hand-off of medications might yield better outcomes. The other trial with novel delivery of varenicline was the COMPASS trial,(71) which tested various iterations of web/phone/proactive counseling. It is presented here because **all trial participants received varenicline through mail without any formal clinical evaluation, as proposed here**. In fact, our eligibility process largely mirrors theirs. Secondary analysis from this trial demonstrated high varenicline adherence,(72) particularly early on, which lends support to our approach. The COMPASS trial did not manipulate; (i.e., test) varenicline sampling per se, but offers guidance and rationale for remote delivery of varenicline as proposed here.

Summary, Significance, & Scientific Premise

None of the studies above, nor our preliminary studies below, would suggest that medication sampling is a panacea for smoking cessation. This is about reach, not efficacy. Sampling is ideally suited for a clinical setting, offering a brief, pragmatic, and behavioral strategy to complement advice to quit. It is meant for all smokers, regardless of their motivation to quit. It requires minimal instruction, either from the person giving it, or to the smoker receiving it. While cessation is of course the ultimate outcome of interest, sampling is primarily about treatment engagement, inducing further use of the medication and inducing quit attempts. **Medication sampling is simply about getting more smokers to use better treatments, sooner**. Strong evidence supports NRT sampling, but whether this translates to varenicline sampling is less clear. It could be the case that medication sampling is only effective for NRT (agonist, OTC availability, minimal side effects & easy dosing), or, as we believe, more generalized and inclusive of wholly different types of cessation medication (agonist/antagonist, Rx status, moderately more side effects, and somewhat more complicated dosing). This is a question worth testing, and testing well. The manufacturer may or may not get OTC status for varenicline, which, either way raises the significance of our study even further. Thus, in sum, varenicline sampling offers at least **tri-fold significance**: 1) **clinical aid to cessation** (broad dissemination of proven cessation agent); 2) **policy implications** (packaging, marketing to broad smokers, including those not ready to quit); and 3) **regulatory consequences** (mimic of potential OTC environment, with minimal clinician involvement).

If this and future trials suggest efficacy of our sampling approach, this would suggest that physicians can and should give out samples of medications to all smokers, regardless of intent to quit. Our NRT sampling work (below) already supports this view, but whether this applies to varenicline is an entirely different story. It would be a leap to suggest that varenicline sampling would compare to NRT sampling, and this is the very reason this proposal offers innovation. Indeed, if varenicline sampling is found not effective to promote quitting, then this would suggest: a) sampling should be restricted to OTC products, b) sampling should be restricted to products that do not have rigid instructions for administration, and/or c) physician guidance is needed for varenicline use. Whether this trial is positive or negative, outcomes will have important influence on clinical practice.

We are now in prime position for a randomized clinical trial within which our goals are to assess the effects of varenicline sampling across a range of cessation related behaviors: a) use and conversion to sustained medication use beyond sampling, b) quit attempts and cessation (7 day point prevalence abstinence), and to c)

evaluate the mechanisms of change: i) confidence,/motivation, ii) autonomy, iii) attitudes towards medication, iv) expectancies. With added comparison group of NRT, we can now make head-to-head comparisons of these two medications on each of these outcomes, even if this comparison is secondary. Sensitivity analyses will examine these outcomes/mechanisms separately for smokers with and without interest in quitting.

Preliminary Studies

NRT Sampling Study 1: Our first trial of NRT sampling (R01 DA021619) recruited smokers who were exclusively unmotivated to quit (N=849) from across the country and randomized them to receive mailed samples of nicotine lozenge or not, and was prominently published in *Archives of Internal Medicine*.(73, 74) Participants in both groups were asked to engage in a practice quit attempt; i.e., merely a brief period of trial abstinence which we believed would remove pressures to quit for good. NRT samples were intended to augment that experience, allowing smokers to see how medications worked and how they could be used. Even among these unmotivated smokers, 73% used the nicotine lozenges given to them. NRT sampling yielded higher rates of quit attempts (43% vs. 34%; RR = 1.3; 95% CI: 1.1-1.5) and marginally higher rates of "floating abstinence" (any 7 day period of non-smoking, ever in study; 19% vs. 15%; RR=1.3; 95% CI: 1.0-1.7). Interestingly, the effect of NRT sampling (vs. not) was greater among African Americans than among Whites, both for inducing quit attempts (OR=2.4 for AA vs. OR=1.5 for White), and for achieving point prevalence abstinence at six month follow-up (OR=3.2 for AA vs. OR=1.2 for White). The race x treatment interaction was not significant, as this study was not adequately powered to detect it. Nonetheless, these widely discrepant effect sizes suggest that NRT sampling could be more effective for underserved populations. Follow-up analyses from this same trial revealed that sampling promoted quitting though many of the pathways shown above. Providing a mere 2 week sample of NRT increased motivation and confidence to quit, increased positive attitudes towards and overall knowledge of NRT, and decreased negative attitudes toward NRT.(75) In fact the effect sizes for these mechanisms were fairly robust (Table 2). Cessation outcomes were modest, and we would never claim that sampling is superior to more intensive treatments. But if a $60 intervention that takes <1 minute to deliver can have any improvement towards this goal, it could have higher impact with wider reach.

Table 2: Changes in Hypothesized Mediators following Medication Sampling

	Pre – Post Change		Effect Size d
	NRT Sampling	Control Group	
Motivation to Quit (range 0-10)	2.0	0.4	.46 (small to intermediate)
Knowledge about NRT (0-10)	2.0	1.0	.42 (small to intermediate)
+ Attitudes Towards NRT (1-4)	0.2	0.0	.5 (intermediate)
- Attitudes Towards NRT (1-4)	- 0.8	0.0	-1.1 (large)

NRT Sampling Study 2: With intramural pilot funding, we followed the above trial with a smaller study (N=157) of smokers across South Carolina.(76) Whereas the above trial tested NRT sampling exclusively among unmotivated smokers, Study 2 explicitly examined the effects of sampling and the role of motivation among three groups of smokers: A) smokers motivated to quit, mailed 2-week samples of both nicotine patch and lozenge, vs. B) unmotivated smokers, given same treatment, vs. C) unmotivated smokers, no treatment. We were particularly interested in Group B, who had shared commonality with both Group A (both received sampling) and Group C (both unmotivated to quit). Over 85% of participants in the two sampling groups used medication. Results after 3 months of follow-up demonstrated that, while initial motivation to quit enhances the likelihood of making an attempt (A >B>C; p<.05), it is not a necessary precursor to quitting success: A~B>C, with ~3-fold increase in abstinence among the two sampling groups, relative to control.

NRT Sampling Study 3: Our latest study of NRT sampling is our most real-world test yet: a cluster randomized clinical trial within primary care settings, across South Carolina (22 clinics; N=1245 participants; 2R01 DA021619).(77) Our focus was on the translational potential of medication sampling as a brief, pragmatic intervention that can be offered to a broad spectrum of smokers within clinical context. Outcomes were impressive, and a manuscript is now in review at a top tier journal. More smokers in the NRT group (vs. control) used cessation medication immediately following the clinic visit (55% vs. 10%; AOR = 12.2; 95% CI: 8.3–18.0). That itself is not that impressive, but the fact that medication usage was persistently higher at six months is (25% vs. 14%; AOR = 2.0; 95% CI: 1.5–2.8). NRT sampling increased the rate of quit attempts in the initial month (24% vs. 18%; AOR = 1.7; 95% CI: 1.1–2.6). Most impressively, self-reported point prevalence abstinence rates were significantly higher within the NRT group at 1- (5% vs2%; AOR = 4.4; 95% CI: 1.8-11.1), 3- (10% vs 5%; AOR = 2.6; 95% CI: 1.6-4.2), and 6-month follow-up (12% vs 8%; AOR = 1.7; 95% CI: 1.1–

2.6). Sensitivity analyses revealed comparable results among smokers who were motivated to quit vs. not, demonstrating that NRT sampling was similarly effective for a broad spectrum of smokers. Despite low absolute rates of all quit outcomes (in line with expectation: see Table 1), there was a robust beneficial treatment effect.

One separate but thematically relevant trial, led by Dr. Cropsey (consultant within; co-author Carpenter), examined more hands on, experiential learning through *in vivo* sampling.(78) We demonstrated that NRT sampling increases adherence for subsequent use, and again demonstrated improvements in attitudinal barriers that undermine use. Collectively, these NRT sampling studies demonstrate: a) the importance and benefits of randomized yet naturalistic medication sampling studies as a pragmatic means to promote quitting, b) the well-established methods to conduct sampling studies, and c) our collective expertise in doing so.

Proof of Concept Varenicline Sampling: Through intramural pilot funds, we have an ongoing pilot study (eventual N=80) to establish feasibility and to get an early sense of effect size. Thus far, 60 smokers have been recruited from across SC (another remote trial) and have been randomized to receive a short supply (identical to proposed here) of varenicline vs. not. Of the 45 smokers (22 control; 23 VRN) to have reached 1-month follow-up, 83% of those in the sampling group are still using varenicline, with self-reported decreases in smoking (7-day CPD avg) far greater than controls (45% vs 14%). About a third (6/19) of those in the sampling group have titrated to 2mg by Week 4, though use has been inconsistent. At 4 weeks, 89% of sampling participants (vs. 56% of control) endorsed the belief that "varenicline can help people quit smoking." While sample size numbers are still small, this experience supports the clinical potential of varenicline sampling, and the importance of rigorous evaluation. We have adequate precautions in place for both pregnancy testing and adverse event monitoring, including potential though unlikely increases in depression and suicidal ideation. Even more importantly, this experience paves the path forward with regard to regulatory oversight, i.e., all procedures proposed herein have already been IRB-approved, allowing us to quicken the startup pace for this proposed trial.

Elsewhere, we have published studies on the attitudinal barriers of medication usage, using both quantitative and qualitative methods,(24, 25) demonstrating eagerness to sample cessation medications, even when smokers hold attitudinal barriers against them. Through our qualitative work, we often heard themes ~'I'd use this medication if a doctor suggested that I should.' Lastly, we have conducted a number of conventional, full-course clinical trials of varenicline. These include trials of varenicline for adolescent smoking cessation (ongoing U01 DA031779), comparisons of varenicline vs. bupropion for adolescent smoking cessation,(79) pilot trials of varencline vs. nicotine patch for adult women,(80) and combination varenicline and N-acetylcysteine for adult smokers.(81) As noted above, Dr. Toll led a trial of varenicline distribution through quitline, and the results guide Finally, Dr. Cropsey is primary mentor of an almost completed F31 award (DA047015) that examines the separate pharmacologic vs. expectancy-related effects of varenicline, which has clear relevance to the proposed trial. In total, we have broad, thorough expertise on the clinical and regulatory issues relevant to varenicline.

II. Specific Aims

We propose a randomized clinical trial with **primary aims** to evaluate the: **1)** use, **2)** consequences (on cessation), and **3)** mechanisms of varenicline sampling. We will recruit between 648-660 smokers and randomize them to receive a 4-week sample of varenicline, NRT, or no sample, and will follow all for 6 months. Our remote focus derives from our hands-off (i.e., translatable) intent, yet our methods include both daily diary assessment and remote biological verification of smoking to enhance rigor. We are mindful of requirements for physician oversight and embed full precautions for safety. Our study sample will include smokers both wanting to quit and not (recruitment of each, with stratified randomization), which will allow for exploratory sensitivity analyses to assess the role of smoker motivation on outcomes.

In accordance with these Aims, we hypothesize that: 1) use of varenicline will be strong: >80% of study sample using medications on at least 50% of the sampling period; 2) relative to non-medication controls, sampling will result in higher incidence of quit attempts and biochemically verified cessation; 3) with greater changes in hypothesized mediators (motivation/confidence, decreases in treatment fatigue). Comparisons of varenicline vs NRT sampling a) uptake, b) mechanisms and c) outcomes are secondary but nonetheless compelling and offer additional significance, innovation, and rigor to our trial.

III. Methods

Our three-fold objective is to determine 1) use, 2) consequences, and 3) mechanisms of varenicline sampling. We propose a three-group, randomized clinical trial in which adult smokers, recruited remotely across SC, inclusive of smokers wanting to quit and not, receive a 2-4 week sample of varenicline, combo NRT, or neither, to use as they wish. Remote, serial assessment of carbon monoxide will be used to verify smoking behavior. <u>Uptake and use</u> will be operationally defined as: a) use of medication (incidence, quantity, duration of use), b) conversion to independent use (i.e., obtaining a prescription for further use), and c) adverse events. We anticipate a side effect profile consistent with other trials of varenicline (some insomnia, nausea) but otherwise benign. <u>Cessation-related outcomes</u> include: a) use of additional quit resources (e.g., calls to quitline), b) quit attempts, and c) cessation (7-day point prevalence abstinence, CO verified). <u>Mechanisms</u> include measures of motivation, confidence, knowledge of and attitudes toward medication, autonomy to quit, expectancies, and cessation fatigue. This is an expanded list from our earlier studies, which allows comparability to NRT sampling but also an opportunity to move forward. Whether and how these non-pharmacologic mechanisms differ for varenicline vs. NRT is an interesting question we can now address, though comparison of mediational models is not our primary focus. An overview of study design is shown in Figure 3.

IIIa Important Design Issues

<u>First</u>, sending prescription medication through mail is not only acceptable, but common in clinical practice. We restrict our recruitment to South Carolina alone given medical oversight from our study physician. Since statewide tobacco policy is a constant (vs. nationwide heterogeneity), this is another advantage of keeping this within South Carolina. <u>Second</u>, we do not capture carbon monoxide levels at baseline (which would also allow us to verify baseline smoking status), and instead rely on self-report of smoking. We recognize this as a limitation, particularly if we wanted to examine levels of CO over time. To address this, we would have to separately send the CO monitor (before baseline assessment) and varenicline (after), which adds unnecessary complexity. CO is used to verify smoking status at follow-up, not to assess changes over time. <u>Third</u>, all participants receive a referral to the SC quitline, with rationale and benefits of use. We believe this is a minimum standard to provide for all smokers. <u>Fourth</u>, our design further extends our progression into technology-based remote assessment. This includes teleconsent, remote CO, and daily diaries during the sampling period. Consistent with translational intent for remote delivery, Week 0 contact will be via phone, but all subsequent follow-up will be online, with adequate precautions in place for safety monitoring. <u>Fifth</u>, our screening procedures now include methods to enhance sample diversity, to increase generalizability. For example, we can ensure that at least 30% of our sample is non-white, and at least 40% are of each gender. Other demographic minimums can be considered (e.g., income, education) if our initial sample is insufficiently representative.

Figure 3: Study Design

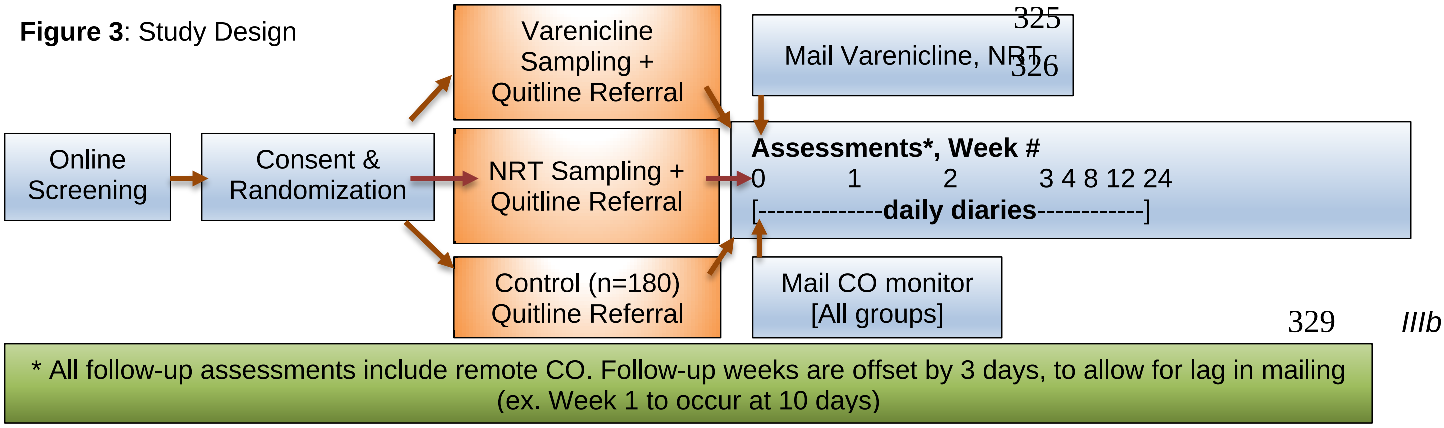

IIIb Participants

As before, we will recruit smokers statewide through various media outlets. Advertising will solicit smokers both motivated to quit and not; this will not be pitched as a treatment study. Smokers will be screened online for study eligibility, which includes innovative and automated methods to protect against duplicate entries, including those who might try to game (falsify) the criteria to gain study access. In our prior statewide sampling

study, we recruited 157 participants in five months, in our ongoing trial of e-cigarettes, we've recruited 375+ in 15 months. Recruitment pace herein might be a bit slower given extra eligibility criteria. We are also excited by our newly developed procedures to ensure sample diversity. Online screening for our remote trials now includes methods to ensure at minimum 40% of each gender, and minimum 30% non-white participants. This is particularly important because a number of eligibility criteria are focused on technological reach (e.g., smartphone ownership), and **these new methods provide greater assurance of sample generalizability**.

a) Eligibility will be contingent upon: 1) age 18+; 2) daily smoker (25+ days per previous month); 3) smoking 5+ cigarettes/day; 4) smoking $\geq$ 1yr; 5) has access to a medical provider who could write a prescription if needed; 6) not currently pregnant, breastfeeding, or planning to become pregnant (remote pregnancy testing is included within our ongoing proof-of-concept study of varenicline, and is working well); 7) no suicidal ideation in past month, nor any lifetime suicide attempt; 8) no current (past month) psychotic symptoms (auditory/visual hallucinations) or self-reported lifetime diagnosis of or treatment for bipolar disorder, schizophrenia, or other psychotic disorders; 9) no medical contraindications for varenicline (e.g., cardiac or renal disease, seizure disorders), 10) own a smartphone (either iOS or Android), and 11) have regular email access. Clinical symptomatology (criteria 7-8) will be based on both the NIMH toolkit for suicidal ideation (5 yes/no items), as well the MINI(82): a] In the past month, have you heard things other people couldn't hear, such as voices? And b] In the past month, have you had visions when you were awake, or have you seen things other people couldn't see? And c] do you have or are you currently in treatment for: schizophrenia, bipolar disorder, or mania. These criteria follow a prior trial that provided varenicline via mail and without direct physician contact.(71) Anyone who screens out on these criteria will receive an individual, separate phone call by Drs. Carpenter, Dahne, Toll, or Gray, be referred elsewhere as needed, and not be included in the study. Our rationale for having access to a medical prescriber (#5 above) is to allow for and promote conversion; so that smokers can readily obtain varenicline after sampling it, if desired. This was unnecessary within our NRT sampling studies, as we just advised participants to seek it out as an OTC product and assessed this as a study outcome. We again assess such conversion in this study but believe it wise to restrict eligibility to those smokers with a means to convert i.e., those with an established healthcare provider. We do not view this as a threat to generalizability, since 70% of smokers visit a physician yearly.(4) Next, although EAGLES trial outcomes documented clear safety (and efficacy) of varenicline even among smokers with comorbid psychiatric disorders, we exclude those with suicidal history and psychosis herein, in an abundance of caution. We require smartphone/email access to allow for daily diary assessment during the sampling period (methods below). If there is a digital divide, it is shrinking. According to latest Pew survey, 81% of Americans own a smartphone, with every demographic >66% (except ages 65+: 53%).(83) Requirement to own smartphone might reduce external validity, but it should not affect internal validity.(84)

IIIc Teleconsent, Baseline Assessment, & Randomization

In our prior studies, recruitment from national sources was based on mail-based return of the consent form. That is, after determination of eligibility, we mailed a consent form (2 copies) and asked the participant to sign one copy and return to us in a pre-addressed, pre-stamped envelope. Receipt on our end is the start of official consent, and official enrollment begins with first phone call (Week 0). While this process generally worked, it was inefficient. For one, mailing delays often meant a lag between the person expressing interest and when s/he was officially enrolled. Second, there are occasional instances where the person does not fully complete the consent form (e.g., missing date), or does not return all necessary pages, and the process repeats itself (back and forth mailing) until we get all forms in full. Third, a large number (~55%) of the consent forms go un-returned, for any number of reasons. Thus, if we want a minimum of 648 participants, we would need to identify ~1180 who would be eligible and interested. Loss of interest in the study is a possibility (which we abide by), but we also believe that consents get lost, thrown away, regarded as junk mail (particularly if seen by someone else), etc. For the current study, we will continue with this procedure of mail-based consenting and add a new teleconsent process that addresses this inefficiency. After the initial determination of study eligibility, assessed online in our secure survey, participants will be asked about their capacity for teleconsent procedures, including access to a device and internet on which they can complete remote consent. For those who have the required hardware and software for teleconsent, we will then describe the basic mechanics of online consent process and ask for their preference for mode of consent: to be consented online or to receive a mailed consent. Participants can choose their preferred method of consent, and none of this is an eligibility

requirement. Participants who do not meet teleconsent requirements will be mailed a consent form following standard procedures. Thus, we are simply offering another, more immediate option for study consent that may be preferable for some participants.

For those who elect teleconsent, we will follow IRB-approved procedures as per precedent. Participants who elect to complete the consent process online using doxy.me or REDCap will be consented by IRB-approved study personnel: the PI, study coordinator, or research assistants. All doxy.me signed consent forms will be saved as .pdf files within our study records and all REDCap documents are immediately saved in REDCap. Most of our ongoing studies employ this method, and a growing number of participants prefer this route of consent (currently ~55%). For those opting to receive consent packet through the mail, we will follow our prior procedures described above. Once they have received the consent form in the mail, we will have a scheduled phone call to discuss the consent and study with them. They will have the opportunity to ask any questions at this time, and participants cannot be enrolled without completing the phone discussion step of the consenting process. This phone call was added to our prior mailed consent procedures to ensure that participants have a full understanding of the consent form before enrolling in the study, as they would in person or with online consent. We have a toll-free phone line to support anyone who calls with questions. Upon return to our office, data are entered into our database, and these individuals comprise the consented sample. However, the sample is reduced further to those with whom we are able to establish first phone contact (Day 0); i.e., the enrolled sample. This enrolled sample is the intent-to-treat sample.

Pre-Randomization Verification of Non-Pregnancy: For females age $\leq$55, we impose one additional step to verify pregnancy status. We will mail these participants a commercially available pregnancy test to verify non-pregnancy status. We will then require these participants to confirm in writing via REDCap that they have completed the pregnancy test and have received a negative result. Participants are also informed that they should let us know if they become pregnant during the trial. Medications will not be sent until this verification is in place. These methods are IRB approved (and required) for our proof-of-concept pilot study.

Pre-Randomization Physician Approval for Medications: Finally, prior to study randomization, our study physician will review a summary of eligibility criteria, collected during Primary (e.g., smoking history, age, medical history and comorbidities) and Secondary screening (psychiatric history, suicidal history, pregnancy status) steps before the participant can be randomized and be enrolled into our trial. We will deliver this summary with a report that we create within our REDCap screening database, to be delivered electronically to the study physician. He will review each individual's information, and will make a determination to: 1) accept the individual into the study, 2) reject the individual into the study (i.e., not eligible for varenicline), or 3) ascertain more information from the individual before either of the decision to accept/reject the participant. In the latter, the study physician may contact the individual to engage in further clinical interview. The disposition of each participant will be tracked within this screening database, allowing us to complete all CONSORT steps to study flow. The individual will not proceed to randomization and Week 0 baseline call until we have signed, explicit documentation from the study physician to do so. This step for pre-randomization physician approval will sequentially follow the pregnancy test evaluation, since the former depends on the latter. Any individuals removed through either pregnancy test (test positive or non-responsive) or through physician discretion will not be counted in the final enrolled study sample. A summary of the overall study flow is depicted in the figure below (following page).

Thus, this procedure ensures that we retain physician oversight and approval of varenicline disbursement at study outset. Our study physician retains his role in evaluation of adverse events as noted elsewhere within this application.

While the source of varenicline within our trial is not yet clear, we will work with a TBD pharmacy to package and label varenicline. Both packaging and labelling will be done within TBD pharmacy, following all applicable laws. Under no circumstances will non-credentialed study staff package or label the medication. The TBD pharmacy will package and label the varenicline under our prescriber's order.

Figure of Study Flow for STARS

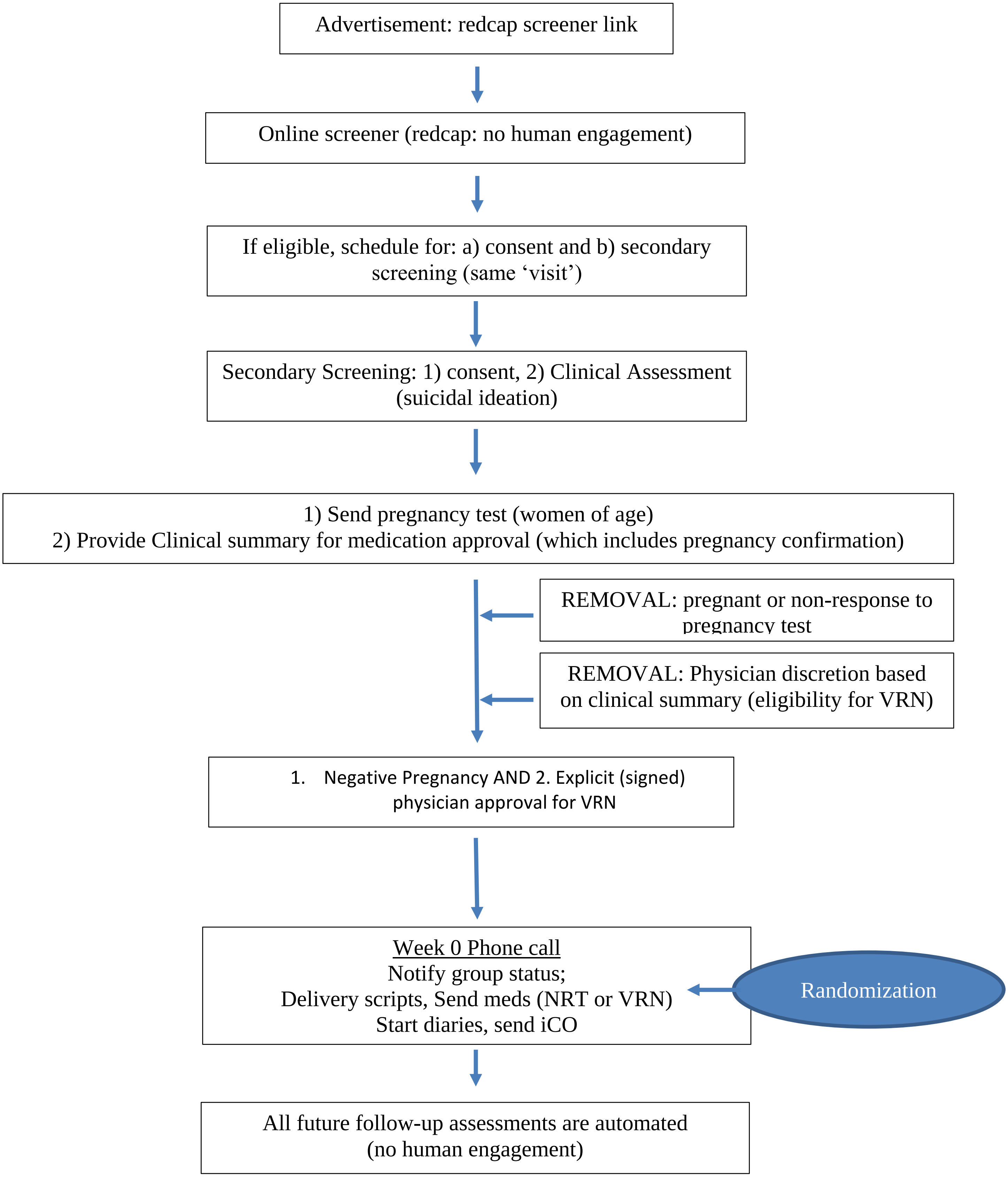

Randomization, Stratification We recruit smokers wanting to quit ($\geq$7 on 0-10 scale) and not, and we use this as a stratification variable. We considered a number of other stratification variables (e.g. cigs/day, insurance status) but believe that randomization will adequately handle these. We will randomize participants to group in a 2:1:1 ratio, with intent to increase precision of our parameter estimates within the varenicline group (n=324), consistent with our intent to understand use patterns and uptake of varenicline.

IIId Interventions and procedures

All smokers in all groups will receive a mailing consisting of standard cessation support materials and specific contact information and benefits of using South Carolina quitline. Quitline benefits will convey the option, for some smokers, to receive free medication. Varenicline participants will receive one-time supply of medication in childproof pill bottle. In our prior sampling studies, we have been largely non-directive of medication use, simply suggesting to participants that they might use it in any number of ways, including not at all. This messaging was consistent with our naturalistic intent, i.e. to allow smokers to self-determine if and how to try the product. For a study of varenicline sampling, we aim to retain naturalistic context but may need to be more directive, including guidance on titration (see dosage comments below). Thus, our messaging will state ~ "*We are offering you a up to a four-week supply of varenicline, which we hope you find helpful. Evidence shows that varenicline is among the best medications to help smokers quit. It is also helpful for smokers who are not yet ready to quit. It is completely up to you if and how you take this. If you decide to try varenicline, start with taking one pill each day for 3 days, followed by a pill in the morning and a pill in the evening thereafter. If you want a stronger dose of medicine to help you quit, you may also go up to two pills in the morning and two pills in the evening after a week. If you ever take a day off and want to re-start, just start this process over again."* This messaging was drafted and refined by four clinicians and is working well in our pilot study above. This messaging will be conveyed directly during intervention call and also within mailing (with accompanying calendar graphics to facilitate understanding). Both the phone-based and mailing messaging of varenicline sampling will suggest options for continued use beyond the sampling period and will be tailored to individual insurance status. As noted above, study eligibility is restricted to smokers who have access to a medical prescriber who can "convert" interested smokers into more sustained varenicline use. Our mailing will also include a generic letter that smokers can give to their provider stating that they have been pre-screened for and have received varenicline and should be considered for further use upon provider approval. It will be up to the participant to decide if and when to offer this letter to their individual provider. We cannot ensure that this conversion will be seamless, i.e., to occur immediately at the end of sampling period. The extent and timing of conversion is one study outcome.

We extensively debated varenicline dosing and sampling duration. Typical practice is to titrate as follows: .5mg QD for days 1-3, .5mg BID for days 4-7, and 1mg BID from day 8 and beyond. On one hand, we want to provide the strongest, most evidence-based dosage possible. If varenicline sampling results in null effect, we do not want this to be due to dosing. On the other hand, providing multiple dosages within a self-paced sampling experience could add unwanted confusion and risk. We are also aware of two large trials that showed similar outcomes of 1mg vs. 2mg dosing, with fewer adverse events in the former.(66, 67) To balance these perspectives, we: a) provide .5mg tablets only, 60 total, b) with instructions to titrate as noted within *italicized instructions* above, c) give instructions to take no more than two in the morning and two in the evening, d) give the participant the ultimate choice to self-pace usage. This choice and pattern of titration becomes an interesting study outcome that aligns with our focus on participant uptake and is wholly consistent with our naturalistic intent. Thus, we view the sampling experience as lasting 2-4 weeks depending on participant choice. For anyone who might ignore the titration schedule and start/restart with 2mg, nausea is the most likely consequence, nothing worse.

Smokers in the NRT group will follow our prior protocol: 4-week supply of both nicotine patch (14mg) and lozenge (4mg), with *comparable instructions for self-determined use as above*. As in our prior trials, we provide both patch and lozenge given different mechanisms of action (slow, cumulative dosing of patch vs. acute delivery for momentary craving with lozenges), and also because of strong evidence to show that superiority of combo NRT over placebo and single NRT products (85-87). We neither explicitly advise nor discourage combined use. Thus, this is an opportunity for smokers to learn about two NRT products, to be used individually or together (participant choice, including none at all). We will caution all participants on signs of receiving too much nicotine (nausea, dizziness) and will monitor adverse events, expected to be rare/mild.

IIIe Follow-Up Procedures

Follow-up assessments will be auto-sent to participants at +17 days (2 weeks beyond initial mailing, accounting for ~ 3 day delay in mailing), +33 days (same delay), +2, +3 and +6 months. Participants are reimbursed for completion of each of five interviews at an escalating rate and with bonus for completion of all. We have excellent history of participant retention across our prior studies. Spanning at least 5 trials, encompassing >10,000 participant phone contacts, we consistently complete 80-90%. In our prior study of NRT sampling in South Carolina, we completed 96% of all scheduled contacts, our best yet. Payments are sent after each completed assessment (gift card or code for online purchase), and again the procedures are well established. Participants will also be able to earn referral bonuses for referring friends and family who enroll in the study.

IIIf Assessments

All interview data are entered in real time within a REDCap database, ensuring high data quality. We have established assessment protocols for all outcomes. Our follow-up interview has been extensively used in our prior studies and is timed at ~20 minutes. Self-report data include the following:

Baseline questionnaire will ascertain basic demographics and smoking history, including measures of dependence,(88) prior use of medications, and all hypothesized mediators (*listed below).

Smoking (Reduction) Using Timeline Follow Back (TLFB) methods, we assess cigarettes per day over the previous seven days, and from this can calculate abstinence (defined below), as well as smoking reduction, and percent who achieve 50% reduction. We also track use of other tobacco products, including e-cigarettes.

Abstinence is defined as both “floating” (any 7 day period of non-smoking) and point prevalence, as recommended for “cessation-induction” studies.(89) We will track longest duration of abstinence and relapse following stated quit attempts.

Quit Attempts We will track the incidence of any self-defined and “serious” quit attempts over the 3-month follow-up period. The latter is the typical measure of quit attempts, used in CDC reports, and is defined as an attempt lasting ≥24hrs. We will also report on the maximum duration, and latency of each QA since baseline.

Use of varenicline, NRT other meds We will determine the incidence and frequency of medications used, both during the sampling period and beyond. For varenicline, we will assess actual dosage used as well as titration patterns, days of use, and whether or not the person “converted” to sustained use (when, how, through whom).

*Self-Efficacy, Motivation, & Confidence to Quit We will use single item (0-10 visual analog scale) measures for each. Our ladders have adequate test-retest stability and predictive validity.(90)

*Attitudes Towards varenicline, NRT Based largely on prior work in this area(58, 91), we have published(25, 75) on 1) positive, and 2) negative attitudes towards medications, as well as 3) general knowledge of them.

*Treatment Self-Regulation Questionnaire The TSRQ(47) is a well-established measure of autonomous motivation; i.e., the degree to which decisions on quitting are self-determined and/or controlled by others.

*Cessation Fatigue We will use a brief measure of hopelessness from our recently developed Cessation Fatigue Scale,(22) which we have subsequently shown to be predictive smoking/relapse.(23)

*Expectancies – We adapt a 6-item measure of medication expectancies from Dr. Toll’s work in bupropion.(92) This adapted version is also used within Dr. Cropsey’s mentee’s F31 (see above).

*Self-Control – We will use a 13-item measure of general self-control, with strong internal consistency and retest reliability.(93)

Depression: We are mindful to assess potential emergence of depressive symptoms, which will be assessed via PHQ9(94). Anyone who reports emergent suicidal ideation within PHQ will be managed by licensed mental health providers (Drs. Carpenter, Dahne, Toll or Gray). Our existing procedures within our proof-of-concept trial include automated data monitoring to identify in real-time any participant who endorses suicidal ideation.

Adverse Events Adverse events will be tracked and rated as mild, moderate or severe by the patient and rated for relatedness to medication by the research assistant using guidelines, with oversight from our study physician. We will determine if any adverse events result in dropouts or are serious according to FDA guidelines.

IIIg Electronic Diaries during Sampling

All participants will be asked to complete a brief diary to assess smoking behavior and use of meds. These diaries, which will include brief mood assessments, provide additional, real-time risk monitoring, not to replace traditional adverse event assessment (above), but to complement. Our intent is to better capture day-to-day fluctuations in medication use and smoking. Each of our recent trials have used this diary approach, with excellent compliance (88% for one study, 83% for another). Diaries will assess cigarettes smoked per day, medications/products used, purposes of that use (to reduce/quit), and mood/affect. Diaries (≤2 min each) will

be electronically collected daily throughout the sampling period (28 days). We have established procedures to auto-send an email/text on a set schedule. Thus, per eligibility criteria above, we require participants to have smartphone capacity, and they will be compensated for compliance based on % diaries completed (e.g., $100 for 90-100% compliance, $70 for 75-90% compliance, etc.). There is some debate as to whether EMA/diary procedures alter behavior, but recent evidence suggests not,(95, 96) but even if so this is equal between groups.

IIIh Remote CO Collection

Our prior remote trials did not include biological verification of smoking. With minimal intensity 'interventions' we view the bias of demand characteristics as low. However, we recognize that biological verification is certainly a strength. MUSC investigators are on the forefront of real-time remote CO collection (K23 DA045766, R34 DA042228, forthcoming NCI R21), in which all participants are provided an iCO Smokerlyzer at the time of enrollment. The device connects to a smartphone (both Android and iOS); thus, we require this as an eligibility criterion. The iCO can detect CO concentrations of 0-100 ppm, and has strong test-retest reliability, as well as concordance with traditional device.(97) Each follow-up will include instructions to complete the iCO breathalyzer, plugging it into their phone to capture video confirmation and all CO readings (time/date stamped). Thus, the procedure provides objective recordings. As of this writing, our group is working on methods to eliminate the need for an app altogether, instead interfacing with REDCap directly (secure, online, real-time data capture); Dr. Dahne has ACS and forthcoming R21 funding explicitly for this purpose. The $70 iCO device can be used repeatedly within a person, but it is not returned at end of study as it is not transferable to another user. A portion of participant compensation is contingent upon iCO compliance (not abstinence). We do not envision iCO testing within each daily diary, but we will add it for any reports of non-smoking, offering an additional method to corroborate quit attempts in real-time, heretofore not possible. These procedures have worked well in pilot testing, but we recognize that they are far from well-established. Alternative options are less feasible. First, we could mail a more established device (piCO), have the person return it after use, and transfer it to another participant. We have investigators at MUSC who are doing so with success. Ostensibly, however, this is no different than above, and allows fewer participants in the trial at one time (infeasible to purchase one device per participant). So, this is not our preferred method. Second, we could send at-home saliva collection kits, and have them returned to our lab for cotinine testing. These methods are more established, yet still unsatisfactory, in terms of compliance, biased responding, timeliness (asynchronized with survey responding), confounds of nicotine (NRT), and cost of assay. In the end, the iCO procedures are imperfect but still the best option available. We define abstinence as 7-day point prevalence (no smoking in prior week), with CO $\leq$4ppm.(98, 99)

IIIi Power Estimations and Data Analyses

Power: Our three-fold focus on outcomes includes 1) uptake (usage, conversion), 2) consequences (quit attempts, abstinence), and 3) mechanisms of varenicline and NRT sampling. We power on the most stringent of these to determine sample size: point-prevalence abstinence at six months (Aim 2), and we do so based on primary comparison of varenicline vs. inactive (non-sampling) control; see rationale above. Based on our early work, and under our belief that varenicline sampling will result in marginally better outcomes than NRT (12-15% in our prior studies) we anticipate a quit rate of 18% in varenicline vs. 8% in control, the latter also in line with our prior studies. With 2:1 randomization for this comparison (VRN:control), and with power of 80%, this will require a total sample size of 413 participants across these two groups. We conservatively inflate this by 15% to account for potential attrition, though in past studies this was closer to 12%, thus resulting in 324 varenicline vs. 162 control participants. We secondarily add another, active comparator group of smokers receiving NRT sampling (n=162; 2:1:1 overall randomization), for a **total of 648 participants**. If we were to power on varenicline vs. NRT outcomes, this would require a total sample of 1300 VRN + 650 NRT (+ 160 possible control), and such a study would be infeasible within this mechanism, and likely infeasible within any R01, particularly given the costs of medication. With anticipated 324 varenicline vs. 162 NRT participants, we have 39% power to detect a difference of 18% vs. 12% in abstinence. We have been doing studies this large for some time and have great confidence in recruitment. Over a 48-month recruitment span (see Timeline below), we aim to recruit ~11/month. For our ongoing nationwide trial of e-cigarettes (eventual N=660), we've averaged ~25/month. Considering the recruitment process and minor time delays between consent and formal enrollment, it is difficult to stop at an exact number. Thus, we will enroll between 648-660 participants to abide by those at earlier steps of the process.

Outcomes pertaining to uptake, usage and conversion (independent use of varenicline, NRT; Aim 1) are focused *within* each active treatment group individually, though we can compare each of these outcomes

between medication groups as well, per binary/continuous analyses below. With 360 total participants in varenicline group, a two-sided 95% confidence interval will have a maximum half-width of 0.052 (e.g., 95% CI on an observed rate of 50% would range from 0.448 to 0.552). With 162 participants in NRT group, the maximum half-width for uptake parameters is .077. As the observed proportion deviates from 50% (either higher or lower), the 95% confidence interval will decrease in width, providing more precision on the estimate (e.g., 95% CI on 90% would range from 0.896 to 0.931 with n=324). A confidence interval as wide as +/- 5% is a relatively small width and one we are willing to accept for our estimates on uptake, and conversion. Formal mediation analyses (Aim 3: mechanisms of change, within and between groups) are not planned, but we can test whether the sampling intervention results in greater change in mechanism variables (*above) similarly vs. differently for varenicline vs. NRT. All analyses are based on an intent-to-treat approach and are nearly identical to analyses from our current and prior studies. Exploratory analysis will be performed on all variables to determine if transformations are needed. Any significant baseline differences between groups will be included in analyses. All analyses will be run separately for full intent-to-treat sample and those retained per protocol. We are specifically interested in whether sampling effects generalize to those who are and are not motivated to quit, and we will examine this via sensitivity (subgroup) analyses. We are under-powered for tests of treatment interactions.

<u>Missing Data & Dropout:</u> The most conservative approach for handling missing data(100) is to substitute them with baseline values, assuming outcomes have all returned to baseline, with no quit attempts made or abstinence achieved. If this assumption is incorrect, it biases the results towards the null by reducing between group differences.(100) We expect few missing data and thus plan for this approach. If missing data >10%, we will calculate results using this conservative method but also using methods in which missing data are imputed as described in the SRNT guidelines, and will assess whether dropout differs by study group (not the case in our prior trials). Abstinence will be reported both with and without (missing samples) CO verification.

<u>Feasibility and Measures of Uptake:</u> Our ongoing proof-of-concept study provides an early sense of study feasibility and thus this is not a main outcome of the trial. Nonetheless, we will deem our approach feasible if: a) at least 80% of varenicline group tries the medication, b) with reasonable duration of use (e.g., median days of use ≥14 days), c) at least 50% of varenicline group self-titrates to 2mg, d) at least 30% convert to independent use, and e) <5% of adverse events from varenicline are serious.

<u>Binary Outcomes</u> (incidence rate of quit attempts & cessation; % treatment uptake/conversion) will first be examined over the entire study period from baseline to end of follow-up by calculating frequencies/percentages as well as 95% confidence intervals for each medication group. Chi-square tests (or Fisher's exact tests as needed) will compare groups on binary outcomes to determine if effects from sampling group exceed those from control group. Both the control group (primary/powered comparison) and NRT group (secondary) will serve as referent for varenicline group outcomes. Covariates will be included where necessary based on baseline differences. All tests will be considered significant at the $\alpha=0.05$ level of significance.

<u>Continuous Outcomes</u> (duration of abstinence, smoking reduction) measured over the entire study period from baseline to the end of follow-up will be evaluated using general linear modeling with a main effect of group to determine group differences. Covariates will be added as necessary to account for baseline group differences.

<u>Mechanisms of Change:</u> We hypothesize that varenicline sampling will enhance quit motivation & confidence, as well knowledge of and attitudes toward medication, autonomy to quit, and will decrease cessation fatigue. We will examine all through analyses of continuous outcomes above. It will be interesting to explore whether and how these non-pharmacologic mechanisms differ for varenicline vs. NRT. Medication expectancies, knowledge, and familiarization might differentially vary through varenicline/NRT sampling, as might motivation and confidence. Note that our focus is on non-pharmacologic mechanisms, since the pharmacologic mechanisms of both varenicline and NRT are well-established and also since our design does not include a placebo comparator. Also note that formal tests of mediation can be explored but we may not be powered for these.

<u>Diary Analyses:</u> Mixed effects regression models(101) will be used to model diary data outcomes (e.g., days of med use) to allow for repeated observations per participant as well as for time-invariant (group, gender, etc.) and time-varying covariates (e.g., motivation to quit). These types of models allow for the inclusion of incomplete data across time, so we can include all reported data, even if <28 days per ppt. Any results obtained from these mixed effects models will be considered exploratory and primarily used to gather information on effect sizes.

Conclusions

In an era when most intensive treatments do not lend themselves to real-world implementation, medication sampling represents a brief, concrete, easy to explain strategy that has strong empirical and theoretical support. Sampling extends the reach of treatment, and thus offers both clinical and policy significance. The proposed varenicline sampling study is not merely a rote extension of our NRT sampling work. Yet like our prior work, we believe that varenicline sampling offers another and potentially stronger catalyst for further engagement in the quitting process. This R01 wholly conforms to recommendations for pragmatic trials,(102, 103) in that our intervention is: rigorous, rapid, transparent, user-friendly, feasible, important to practitioners, and eventually broadly applicable.(104, 105) This application is also wholly responsive to PAR 18-869, and, as a modular R01, offers a tight, streamlined clinical trial that directly tobacco control, which is cancer control. Varenicline sampling, applied to a broad spectrum of smokers, could be yet another strategy to get more smokers to make evidence-based quit attempts, sooner.

REFERENCES

1. USDHS. The Health Consequences of Smoking - 50 Years of Progress: A Report of the Surgeon General. Washington, DC: US Govt. Printing Office; 2014.
2. Xu X, Bishop EE, Kennedy SM, Simpson SA, Pechacek TF. Annual healthcare spending attributable to cigarette smoking. American Journal of Preventive Medicine. 2015;48:326-33.
3. Amodei N, Lamb RJ. Over-the-counter nicotine replacement therapy: Can its impact on smoking cessation be enhanced? Psychology of Addictive Behaviors. 2008;22:472-85.
4. Fiore MC, Jaen CR, Baker TB, Bailey WC, Benowitz NL, Curry SJ, et al. Treating tobacco use and dependence: 2008 Update. Clinical Practice Guideline. Rockville, MD: US Public Health Service; 2008.
5. Silagy C, Lancaster T, Stead L, Mant D, Fowler G. Nicotine replacement therapy for smoking cessation 2004 [Issue 3:[
6. Stead LF, Bergson G, Lancaster T. Physician advice for smoking cessation (Cochrane Review). The Cochrane Library, Issue 3. Oxford: Wiley Publishers; 2008.
7. Stead LF, Lancaster T. Telephone counselling for smoking cessation (Cochrane Review). The Cochrane Library, Issue 3. Oxford: Updated Software; 2001.
8. Villanti AC, McKay HS, Abrams DB, Holtgrave DR, Bowie JV. Smoking-cessation interventions for U.S. young adults: A systematic review. American Journal of Preventive Medicine. 2010;39(6):564-74.
9. Dahne J, Wahlquist AE, Garrett-Mayer E, Heckman BW, Cummings KM, Carpenter MJ. State tobacco policies as predictors of evidence-based cessation method usage: Results from a large, nationally representative dataset. Nicotine & Tobacco Research. 2018;20:1336-43.
10. Dahne J, Wahlquist AE, Garrett-Mayer E, Heckman BW, Cummings KM, Carpenter MJ. The differential impact of state tobacco control policies on cessation treatment utilization across established tobacco disparities groups. Preventive Medicine. 2017;105:319-25.
11. Fix BV, Hyland A, Rivard C, McNeill A, Fong GT, Borland R, et al. Usage patterns of stop smoking medications in Australia, Canada, the United Kingdom, and the United States: Findings from the 2006-2008 International Tobacco Control (ITC) Four Country Survey. International Journal of Environmental Research and Public Health. 2011;8(1):222-33.
12. Willems RA, Willemsen MC, Nagelhout GE, De Vries H. Understanding smokers' motivations to use evidence-based smoking cessation aids. Nicotine & Tobacco Research. 2013;15:167-76.
13. Cook-Shimanek M, Burns EK, Levinson AH. Medicinal nicotine nonuse: Smokers' rationales for past behavior and intentions to try medicinal nicotine in a future quit attempt. Nicotine & Tobacco Research. 2013;15:1926-33.
14. Hines D. Young smokers' attitudes about methods for quitting smoking: Barriers and benefits to using assisted methods. Addictive Behaviors. 1996;21:531-5.
15. Shiffman S, Brockwell SE, Pillitteri JL, Gitchell JG. Individual differences in adoption of treatment for smoking cessation: Demographic and smoking history characteristics. Drug Alcohol Depend. 2008;93(1-2):121-31.
16. Shiffman S, Brockwell SE, Pillitteri JL, Gitchell JG. Use of smoking-cessation treatments in the United States. Am J Prev Med. 2008;34(2):102-11.
17. Borland R, Cooper J, McNeill A, O'Connor R, Cummings KM. Trends in beliefs about the harmfulness and use of stop-smoking medications and smokeless tobacco products among cigarettes smokers: Findings from the ITC four-country survey. Harm Reduction Journal. 2011;8.
18. Caraballo RS, Shafer PR, Patel D, Davis KC, McAfee TA. Quit Methods Used by US Adult Cigarette Smokers, 2014–2016. Preventing Chronic Disease 2017;14.

19. Baker TB, Breslau N, Covey L, Shiffman S. DSM criteria for tobacco use disorder and tobacco withdrawal: A critique and proposed revisions for DSM-5. Addiction. 2012;107:263-75.
20. Chaiton M, Diemert L, Cohen JE, Bondy SJ, Selby P, Philipneri A, et al. Estimating the number of quit attempts it takes to quit smoking successfully in a longitudinal cohort of smokers. BMJ Open. 2016;6:e011045.
21. Partos TR, Borland R, Yong HH, Hyland A, Cummings KM. The quitting rollercoaster: How recent quitting history affects future cessation outcomes (data from the International Tobacco Control 4-Country Cohort Study). Nicotine & Tobacco Research. 2013;15:1578-87.
22. Mathew AR, Heckman BW, Meier E, Carpenter MJ. Development and initial validation of a cessation fatigue scale. Drug and Alcohol Dependence. 2017;176:102-8.
23. Heckman BW, Dahne JR, Mathew AR, Germeroth LJ, Santa Ana EJ, Saladin ME, et al. Does cessation fatigue predict smoking-cessation milestones? A longitudinal study of current and former smokers. Journal of Consulting and Clinical Psychology. 2018;86:903-14.
24. Carpenter MJ, Ford ME, Cartmell KB, Alberg AJ. Misperceptions of nicotine replacement therapy within racially and ethnically diverse smokers. Journal of the National Medical Association. 2011;103:885-94.
25. Ryan KK, Garrett-Mayer E, Alberg AJ, Cartmell KB, Carpenter MJ. Predictors of cessation pharmacotherapy use among Black and non-Hispanic white smokers. Nicotine & Tobacco Research. 2011;13:646-52.
26. Mooney ME, Leventhal AM, Hatsukami DK. Attitudes and knowledge about nicotine and nicotine replacement therapy. Nicotine & Tobacco Research. 2006;8:435-46.
27. Vogt F, Hall S, Marteau TM. Understanding why smokers do not want to use nicotine dependence medications to stop smoking: Qualitative and quantitative studies. Nicotine & Tobacco Research. 2008;10:1405-13.
28. Hughes JR, Marcy TW, Naud S. Interest in treatments to stop smoking. Journal of Substance Abuse Treatment. 2009;36:18-24.
29. Fu SS, Sherman SE, Yano EM, van Ryn M, Lanto AB, Joseph AM. Ethnic disparities in the use of nicotine replacement therapy for smoking cessation in an equal access health care system. American Journal of Health Promotion. 2005;20:108-16.
30. Levinson AH, Borrayo EA, Espinoza P, Flores ET, Pérez-Stable EJ. An exploration of latino smokers and the use of pharmaceutical aids. American Journal of Preventive Medicine. 2006;31:167-71.
31. Muraven M, Baumeister RF. Self-regulation and depletion of limited resources: Does self-control resemble a muscle? Psychological Bulletin. 2000;126:247-59.
32. Muraven M, Slessareva E. Mechanisms of self-control failure: Motivation and limited resources. Personality and Social Psychology. 2003;29:894-906.
33. Muraven M. Practicing self-control lowers the risk of smoking lapse. Psychology of Addictive Behaviors. 2010;24:446-52.
34. USDHS. Current cigarette smoking among adults - United States, 2005-2015. MMWR. 2016;65:1205-11.
35. Fiore MC, McCarthy DE, Jackson TC, Zehner ME, Jorenby DE, Mielke M, et al. Integrating smoking cessation treatment into primary care: An effectiveness study. Preventive Medicine. 2004;38:412-20.
36. Cunningham JA, Kushnir V, McCambridge J. The impact of asking about interest in free nicotine patches on smoker's stated intent to change: real effect or artefact of question ordering? Nicotine & Tobacco Research. 2016;18:1215-7.
37. Walker N, Howe C, Bullen C, Grigg M, Glover M, McRobbie H, et al. Does improved access and greater choice of nicotine replacement therapy affect smoking cessation success? Findings from a randomised controlled trial. Addiction. 2011;106(6):1176-85.
38. Cummings KM, Fix BV, Celestino P, Hyland A, Mahoney M, Ossip DJ, et al. Does the number of free nicotine patches given to smokers calling a quitline influence quit rates: Results from a quasi-experimental study. BMC Public Health. 2010;10:181.
39. McAfee TA, Bush T, Deprey TM, Mahoney LD, Zbikowski SM, Fellows JL, et al. Nicotine patches and uninsured quitline callers. A randomized trial of two versus eight weeks. American Journal of Preventive Medicine. 2008;35(2):103-10.
40. Tinkelman D, Wilson SM, Willett J, Sweeney CT. Offering free NRT through a tobacco quitline: Impact on utilisation and quit rates. Tobacco Control. 2007;16(Suppl 1):i42-i6.

41. Schillo BA, Wendling A, Saul J, Luxenberg MG, Lachter R, Christenson M, et al. Expanding access to nicotine replacement therapy through Minnesota's QUITLINE partnership. Tobacco Control. 2007;16(Suppl 1):i37-i41.
42. Hollis JF, McAfee TA, Fellows JL, Zbikowski SM, Stark M, Riedlinger K. The effectiveness and cost effectiveness of telephone counselling and the nicotine patch in a state tobacco quitline. Tobacco Control. 2007;16(Suppl 1):i53-i9.
43. Cummings KM, Hyland A, Carlin-Menter S, Mahoney MC, Willett J, Juster HR. Costs of giving out free nicotine patches through a telephone quit line. Journal of Public Health Management and Practice. 2011;17(3):E16-23.
44. Godin G, Valois P, L. L, Desharnais R. Predictors of smoking behaviour: An application of Ajzen's Theory of Planned Behaviour. British Journal of Addiction. 1992;87:1335-43.
45. Norman P, Conner M, Bell R. The Theory of Planned Behavior and smoking cessation. Health Psychology. 1999;18:89-94.
46. Orbell S, Hagger M, Brown V, Tidy J. Comparing two theories of health behavior: A prospective study of noncompletion of treatment following cervical cancer screening. Health Psychology. 2006;25:604-15.
47. Williams GC, Niemiec CP, Patrick H, Ryan RM, Deci EL. The importance of supporting autonomy and perceived competence in facilitating long-term tobacco abstinence Annals of Behavioral Medicine. 2009;37:315-24.
48. Williams GC, McGregor HA, Zeldman A, Freedman ZR, Deci EL. Testing a self-determination theory process model for promoting glycemic control through diabetes self-management Health Psychology. 2004;23:58-66.
49. Garrison GD, Dugan SE. Varenicline: A first-line treatment option for smoking cessation. Clinical Therapeutics. 2009;31:463-91.
50. Gibbons RD, Mann JJ. Varenicline, smoking cessation, and neuropsychiatric adverse events. American Journal of Psychiatry. 2013;170:1460-7.
51. Cahill K, Stevens S, Perera R, Lancaster T. Pharmacological interventions for smoking cessation: An overview and network meta-analysis. Cochrane Database of Systematice Reviews. Oxford: John Wiley & Sons; 2013.
52. Gonzales D, Rennard SI, Nides M, Oncken C, Azoulay S, Billing CB, et al. Varenicline, an alpha4 beta2 nicotinic acetylcholine receptor partial agonist, vs sustained-release bupropion and placebo for smoking cessation: A randomized controlled trial. JAMA. 2006;296:47-55.
53. Jorenby DE, Hays JT, Rigotti NA, Azoulay S, Watsky EJ, Williams KE, et al. Efficacy of varenicline, an alpha beta2 nicotinic acetylcholine receptor partial agonist, vs placebo or sustained-release bupropion for smoking cessation: A randomized controlled trial. JAMA. 2006;296:56-63.
54. Rollema H, Coe JW, Chambers LK, Hurst RS, Stahl S, M,, Williams KE. Rationale, pharmacology and clinical efficacy of partial agonists of alpha4beta2 nACh receptors for smoking cessation. Trends in Pharmacology Science. 2007;28:316-25.
55. Jiménez-Ruiz C, Berlin I, Hering T. Varenicline: A novel pharmacotherapy for smoking cessation. Drugs. 2009;69:1319-38.
56. West R, Eden Evins A, Benowitz NL, Russ C, McRae T, Lawrence D, et al. Factors associated with the efficacy of smoking cessation treatments and predictors of smoking abstinence in EAGLES. Addiction. 2018;113:1507-16.
57. Anthenelli RM, Benowitz NL, West R, St Aubin L, McRae T, Lawrence D, et al. Neuropsychiatric safety and efficacy of varenicline, bupropion, and nicotine patch in smokers with and without psychiatric disorders (EAGLES): A double-blind, randomised, placebo-controlled clinical trial. Lancet. 2016;387:2507-20.
58. Etter JF, Schneider NG. An internet survey of use, opinions and preferences for smoking cessation medications: nicotine, varenicline, and bupropion. Nicotine & Tobacco Research. 2013;15:59-68.
59. Hughes JR. Varenicline as a cause of suicidal outcomes. Nicotine & Tobacco Research. 2016;18:2-9.
60. Kotz D, Simpson C, Viechtbauer W, van Schayck OC, West R, Sheikh A. Cardiovascular and neuropsychiatric safety of varenicline and bupropion compared with nicotine replacement therapy for smoking cessation: Study protocol of a retrospective cohort study using the QResearch general practice database. BMJ Open. 2014;4.

61. Kotz D, Viechtbauer W, Simpson C, van Schayck OC, West R, Sheikh A. Cardiovascular and neuropsychiatric risks of varenicline: A retrospective cohort study. The Lancet Respiratory Medicine. 2015;10:761-8.

62. Prochaska JJ, Hilton JF. Risk of cardiovascular serious adverse events associated with varenicline use for tobacco cessation: systematic review and meta-analysis. BMJ (Clinical Research Ed). 2012;344:e2856.

63. Ebbert JO, Hughes JR, West RJ, Rennard SI, Russ C, McRae TD, et al. Effect of varenicline on smoking cessation through smoking reduction: A randomized clinical trial. JAMA. 2015;313:687-94.

64. Ebbert JO, Croghan IT, North F, Schroeder DR. A pilot study to assess smokeless tobacco use reduction with varenicline. Nicotine & Tobacco Research. 2010;12(10):1037-40.

65. Rennard S, Hughes J, Cinciripini PM, Kralikova E, Raupach T, Arteaga C, et al. A randomized placebo-controlled trial of varenicline for smoking cessation allowing flexible quit dates. Nicotine & Tobacco Research. 2012;14:343-50.

66. Oncken C, Gonzales D, Nides M, Rennard S, Watsky E, Billing CB, et al. Efficacy and safety of the novel selective nicotinic acetylcholine receptor partial agonist, varenicline, for smoking cessation. Archives of Internal Medicine. 2006;166:1571-7.

67. Fouz-Rosón N, Montemayor-Rubio T, Almadana-Pacheco V, Montserrat-García S, Gómez-Bastero AP, Romero-Muñoz C, et al. Effect of 0.5 mg versus 1 mg varenicline for smoking cessation: a randomized controlled trial. Addiction. 2017;112:1610-9.

68. Biazzo LL, Froshaug DB, Harwell TS, Beck HN, Haugland C, Campbell SL, et al. Characteristics and abstinence outcomes among tobacco quitline enrollees using varenicline or nicotine replacement therapy. Nicotine & Tobacco Research. 2010;12:567-73.

69. Rojewski AM, A. H, Mahoney MC, L.M. C, Zuromski KL, Celestino P, et al. Feasibility of delivering varenicline through a telephone quitline to promote smoking cessation. Journal of Smoking Cessation. 2018;13:201-6.

70. Selby P, Hussain S, Voci S, Zawertailo L. Empowering smokers with a web-assisted tobacco intervention to use prescription smoking cessation medications: A feasibility trial. Implementation Science. 2015;10:139.

71. Swan GE, McClure JB, Jack LM, Zbikowski SM, Javitz HS, Catz SL, et al. Behavioral counseling and varenicline treatment for smoking cessation. American Journal of Preventive Medicine. 2010;38:482-90.

72. Catz SL, Jack LM, McClure JB, Javitz HS, Deprey M, Zbikowski SM, et al. Adherence to varenicline in the COMPASS smoking cessation intervention trial. Nicotine & Tobacco Research. 2011;13(5):361-8.

73. Carpenter MJ, Alberg AJ, Gray KM, Saladin ME. Motivating the unmotivated for health behavior change: A randomized trial of cessation induction for smokers. Clinical Trials. 2010;7:157-66.

74. Carpenter MJ, Hughes JR, Gray KM, Wahlquist AE, Saladin ME, Alberg AJ. Nicotine therapy sampling to induce quit attempts among smokers unmotivated to quit: A randomized clinical trial. Archives of Internal Medicine. 2011;171:1901-7.

75. Burris JL, Heckman BW, Mathew AR, Carpenter MJ. A mechanistic test of nicotine replacement therapy sampling for smoking cessation induction. Psychology of Addictive Behaviors. 2015;29:392-9.

76. Jardin BF, Cropsey KL, Wahlquist AE, Gray KM, Silvestri GA, Cummings KM, et al. Evaluating the effect of access to free medication to quit smoking: A clinical trial testing the role of motivation. Nicotine & Tobacco Research. 2014;16:992-9.

77. Dahne J, Wahlquist AE, Boatright AS, Garrett-Mayer E, Fleming DO, Davis R, et al. Nicotine replacement therapy sampling via primary care: Methods from a pragmatic cluster randomized clinical trial. Contemporary Clinical Trials. 2018;72:1-7.

78. Cropsey KL, Hendricks PS, Schiavon S, Sellers A, Froelich M, Shelton RC, et al. A pilot trial of in vivo NRT sampling to increase medication adherence and abstinence in community corrections smokers. Addictive Behaviors. 2017;67:92-9.

79. Gray KM, Carpenter MJ, Lewis AL, Klintworth EM, Upadhyaya HP. Varenicline versus bupropion XL for smoking cessation in older adolescents: A randomized, double-blind pilot trial. Nicotine & Tobacco Research. 2012;14:234-9.

80. Gray KM, McClure EA, Baker NL, Hartwell KJ, Carpenter MJ, Saladin ME. An exploratory short-term double-blind randomized trial of varenicline versus nicotine patch for smoking cessation in women. Addiction. 2015;110:1027-34.

81. McClure EA, Baker NL, Gipson CD, Carpenter MJ, Roper AP, Froeliger BE, et al. An open-label pilot trial of N-acetylcysteine and varenicline in adult cigarette smokers. American Journal of Drug and Alcohol Abuse. 2015;41:52-6.
82. Sheehan DV, Lecrubier Y, Sheehan KH, Amorim P, Janavs J, Weiller E, et al. The Mini-International Neuropsychiatric Interview (M.I.N.I.): The development and validation of a structured diagnostic psychiatric interview for DSM-IV and ICD-10. Journal of Clinical Psychiatry. 1998;59:22-33.
83. Pew Research Center. Mobile Fact Sheet 2019 [updated June 12, 2019. https://www.pewinternet.org/fact-sheet/mobile/].
84. Etter JF, Perneger TV. A comparison of cigarette smokers recruited through the internet or by mail. International Journal of Epidemiology. 2001;30:521-5.
85. Smith SS, McCarthy DE, Japuntich SJ, Christiansen B, Piper ME, Jorenby DE, et al. Comparative effectiveness of 5 smoking cessation pharmacotherapies in primary care clinics. Archives of Internal Medicine. 2009;14:2148-55.
86. Piper ME, Smith SS, Schlam TR, Fiore MC, Jorenby DE, Fraser D, et al. A randomized placebo-controlled clinical trial of 5 smoking cessation pharmacotherapies. Archives of General Psychiatry. 2009;66:1253-62.
87. Carpenter MJ, Jardin BF, Burris JL, Mathew AR, Schnoll RA, Rigotti NA, et al. Clinical strategies to enhance the efficacy of nicotine replacement therapy for smoking cessation: A review of the literature. Drugs. 2013;73:407-26.
88. Borland R, Yong HH, O'Connor RJ, Hyland A, Thompson ME. The reliability and predictive validity of the Heaviness of Smoking Index and its two components: findings from the International Tobacco Control Four Country study. Nicotine & Tobacco Research. 2010(Supp 1):S45-S50.
89. Aveyard P, Wang D, Connock M, Fry-Smith A, Barton P, Moore D. Assessing the outcomes of prolonged cessation-induction and aid-to-cessation trials: Floating prolonged abstinence. Nicotine & Tobacco Research. 2009;11:475-80.
90. Carpenter MJ, Hughes JR, Solomon LJ, Callas PW. Both smoking reduction with nicotine replacement therapy and motivational advice increase future cessation among smokers unmotivated to quit. Journal of Consulting and Clinical Psychology. 2004;72:371-81.
91. Hammond D, McDonald PW, Fong GT, Borland R. Do smokers know how to quit? Knowledge and perceived effectiveness of cessation assistance as predictors of cessation behaviour. Addiction. 2004;99:1042-8.
92. Fucito LM, Toll BA, Salovey P, O'Malley SS. Beliefs and attitudes about bupropion: Implications for medication adherence and smoking cessation treatment. Psychology of Addictive Behaviors. 2009;23:373-9.
93. Tangney JP, Baumeister RF, Boone AL. High self-control predicts good adjustment, less pathology, better grades, and interpersonal success. Journal of Personality. 2004;72:271-324.
94. Martin A, Rief W, Klaiberg A, Braehler E. Validity of the Brief Patient Health Questionnaire Mood Scale (PHQ-9) in the general population. General Hospital Psychiatry. 2006;28:71-7.
95. McCarthy DE, Minami H, Yeh VM, Bold KW. An experimental investigation of reactivity to ecological momentary assessment frequency among adults trying to quit smoking. Addiction. 2015;110(10):1549-60.
96. Tomko RL, Gray KM, Oppenheimer SR, Wahlquist AE, McClure EA. Using REDCap for ambulatory assessment: Implementation in a clinical trial for smoking cessation to augment in-person data collection. American Journal of Drug and Alcohol Abuse. In Press:1-16.
97. McClure EA, Tomko RL, Carpenter MJ, Treiber FA, Gray KM. Acceptability and compliance with a remote monitoring system to track smoking and abstinence among young smokers. American Journal of Drug and Alcohol Abuse. 2018;44:561-70.
98. Cropsey KL, Trent LR, Clark CB, Stevens EN, Lahti AC, Hendricks PS. How low should you go? Determining the optimal cutoff for exhaled carbon monoxide to confirm smoking abstinence when using cotinine as reference. Nicotine and Tobacco Research. 2014;16:1348-55.
99. Perkins KA, Karelitz JL, Jao NC. Optimal carbon monoxide criteria to confirm 24-hr smoking abstinence. Nicotine & Tobacco Research. 2013;15:978-82.
100. Hall SM, Delucchi KL, Velicer WF, Kahler CW, Ranger-Moore J, Hedeker D, et al. Statistical analysis of randomized trials in tobacco treatment: Longitudinal designs with dichotomous outcome. Nicotine Tob Res. 2001;3:193-202.

101. Hedeker D, Mermelstein RJ. Mixed-effects regression models with heterogeneous variance: Analyzing ecological momentary assessment (EMA) data. In: Little TD, Bovaird JA, Card NA, editors. Modeling Contextual Effects in Longitudinal Studies. Mahwah, NJ: Erlbaum; 2007. p. 183-206.
102. Glasgow RE, Lichtenstein E, Marcus AC. Why don't we see more translation of health promotion research to practice? Rethinking the efficacy-to-effectiveness transition. American Journal of Public Health. 2003;93:1261-7.
103. Treweek S, Zwarenstein M. Making trials matter: Pragmatic and explanatory trials and the problem of applicability. Trials. 2009;10:37.
104. Glasgow RE. What does it mean to be pragmatic? Pragmatic methods, measures, and models to facilitate research translation. Health Education and Behavior 2013;40:257-65.
105. Glasgow RE, Magid DJ, Beck A, Ritzwoller D, Estabrooks PA. Practical clinical trials for translating research to practice: design and measurement recommendations. Medical Care. 2005;43:551-7.

Protocol Amendments: 31 amendments in total, all listed below. Details provided for all amendments that are NOT any of the following: a) change in study personnel, b) change in study advertising, or c) change in study assessments. All amendments received expedited (i.e., not full board) review.

Amend. #	Name	Date
31	Amendment 31 for IRB Study #Pro00098479 –<br>• Staff change	5/28/2025
30	Amendment 30 for IRB Study #Pro00098479 –<br>• Fixing protocol version date	10/14/2024
29	Amendment 29 for IRB Study #Pro00098479 –<br>• Increasing upper limit of study sample from 648 to 660	09/13/2024
28	Amendment 28 for IRB Study #Pro00098479 –<br>• Staff change	5/31/2024
27	Amendment 27 for IRB Study #Pro00098479 –<br>• Staff change	04/22/2024
26	iCO window change<br>• Informed Consent:<br>Changing the breathalyzer sample submission window from 24 hours to 72 hours.	03/12/2024
25	Amendment 25 for IRB Study #Pro00098479<br>• advertising	03/07/2024
24	Amendment 24 for IRB Study #Pro00098479<br>• Staff change	1/19/2024
23	Increase iCO payment<br>• Payment to participant language has been updated to reflect increase in breathalyzer sample from $5 per sample to $20 per sample. Total amount of payment for participants who complete all aspects of the study increased from $225 to $300.	12/20/2023
22	new ads ONLY (from Build Clinical)<br>• New advertising	11/14/2023
21	Personnel Add Only<br>• Staff change	09/15/2023
20	Personnel Add Only<br>• Staff change	06/12/2023
19	Radio Ad<br>• Adding advertisement through radio ad commercials.	03/28/2023
18	New advertisements & addition of referral recruitment<br>• New advertising	02/21/2023
17	New Advertisements Only<br>• Adding additional advertisement photos, flyers, and language.	09/30/2022
16	New ads, remove personnel & removal of 50/50 split based on motivation to quit<br>• new advertisements	07/25/2022

	• We are removing the requirement to recruit equal numbers of "motivated" and "unmotivated" smokers. We will continue to recruit from both groups and continue to stratify on this variable but will no longer exclude participants based on this criteria.<br>• Staffing change	
15	New advertisements only<br>• Three new study flyers	06/22/2022
14	Add 2nd generic varenicline option; personnel addition<br>• Add new generic varenicline (PAR) to ICD<br>• New personnel	06/03/2022
13	Personnel Add Only<br>• Staff change	05/23/2022
12	Personnel Add Only<br>• Staff change	03/04/2022
11	Change medical record language on ICD<br>• Sending study invitations to potential participants through EHR.	02/25/2022
10	Addition of one advertising image & personnel changes<br>• Updated advertising<br>• Staffing change	02/08/2022
9	Changes to VRN source and bus ads<br>• Consent form has been adjusted to reflect the option of giving the FDA authorized generic version of varenicline called Apo-varenicline that is approved and available for use in Canada. Until Chantix becomes available again in the US, we will offer Apo-varenicline in the study.<br><br>We had previously adjusted the script for those in the varenicline group and created a letter to send out to currently enrolled participants who have received varenicline to inform them of a voluntary recall of varenicline at the warehouse level due to impurities found during quality control testing. We now have removed these changes and have made the changes above to the consent form. We will move forward with fda authorized apo-varenicline or chantix once it becomes available again without impurities.	08/11/2021
8	Changing Requirements RE: Having a doctor<br>• Removing the requirement for participants to have a doctor that can prescribe them medications.<br>• Adding a mailed handout to give to ppts with instructions on how to have a telehealth visit for a prescription.	06/30/2021
7	Amendment 7 for IRB Study #Pro00098479 -- Epic Recruitment<br>• Recruitment through EPIC (EHR)<br>• Staffing change	06/23/2021
6	Amendment 6 for IRB Study #Pro00098479 (New Ad Wording)<br>• Revisions to advertising	05/17/2021
5	Amendment 5 for IRB Study #Pro00098479 (New Ads)<br>• New Flyers for Study Recruitment<br>• Staff change	05/10/2021
4	Amendment 4 for IRB Study #Pro00098479 (Corrections to consent form and changed screening questions)<br>• Fixed typos in consent form,<br>adjusted screening/eligibility question from "Have you seen a doctor in the past year?" to " Do you have access to a medical provider who could write a prescription for you if you needed it?"	04/07/2021
3	Amendment 3 for IRB Study #Pro00098479 (Eligibility corrected and assessment changes)<br>• Assessments have been reorganized based on the needs of the study at different time points.<br>iCO collection survey added.<br>Correcting eligibility to require email for all participants (instead of email OR smartphone)	02/18/2021

2	Amendment 2 for IRB Study #Pro00098479<br>• Adding questions to the demographics form for physician review.<br>• Adding DOB to baseline questionnaires.<br>• Adjusting consent form to clarify payment cut offs.<br>• Staffing change	01/22/2021
1	Amendment 1 for IRB Study #Pro00098479<br>• Assessments adjusted for clarity<br>• PHQ cutoff for intervention adjusted - in protocol<br>• Consent form adjusted to allow us to use screening data in the study	09/30/2020
