# Supplementary material for: Medication Samples and Smoking Cessation Among Adults: A Randomized Clinical Trial
Source: JAMA Netw Open. 2026 May 8;9(5):e2611418. doi: 10.1001/jamanetworkopen.2026.11418 (PMC13156786; doi:10.1001/jamanetworkopen.2026.11418)
Supplement: Supplement 2. — eAppendix 1. Treatment Materials Within Medication Sampling Groups eAppendix 2. Alternative Models, With and Without Adjustment eAppendix 3. CO Adherence With Self-Reported Abstinence, per Group, per Time Point eAppendix 4. Treatment Mechanisms eAppendix 5. Analyses by Motivation to Quit eAppendix 6. Adverse Events [file jamanetwopen-e2611418-s002.pdf]

Supplemental Online Content

Carpenter MJ, Smith TT, Wahlquist AE, et al. Medication samples and smoking cessation among adults who smoke: a randomized clinical trial. *JAMA Netw Open.* 2026;9(5):e2611418. doi:10.1001/jamanetworkopen.2026.11418

eAppendix 1. Treatment Materials Within Medication Sampling Groups

eAppendix 2. Alternative Models, With and Without Adjustment

eAppendix 3. CO Adherence With Self-Reported Abstinence, per Group, per Time Point

eAppendix 4. Treatment Mechanisms

eAppendix 5. Analyses by Motivation to Quit

eAppendix 6. Adverse Events

This supplemental material has been provided by the authors to give readers additional information about their work.

© 2026 Carpenter MJ et al. *JAMA Network Open*.

eAppendix 1. Treatment Materials Within Medication Sampling Groups
Varenicline messaging, both oral and written/mailed

Oral: *Baseline Phone Call / Group Assignment*
You have been assigned to the group that will receive the sample of varenicline. Sometimes called Chantix, varenicline is a prescription medication that helps smokers quit smoking. It both reduces cravings for and decreases the pleasurable effects of cigarettes and other tobacco products. It does not contain nicotine. Studies consistently show that smokers who use varenicline have a greater chance of quitting smoking. Even if you're not ready to quit now, varenicline also helps smokers reduce how much they smoke. In fact, most guidelines and medical organizations consider varenicline to be the best single medication available to help smokers quit. There are three important things you need to know about varenicline.

> First, varenicline is a prescription medication, which usually means that you need to see a doctor to get it. In this study, varenicline will be prescribed by our study physician. We do encourage you to talk to your doctor about this study, and about ways to quit smoking. We will give you a letter that you can give to your doctor to start this conversation.
>
> Second, although varenicline is safe for use, some side effects are possible. The most common of these are nausea and insomnia. But the overall safety of varenicline is well-established.
>
> Third, smokers in our study will be given varenicline free of charge; there will be no cost to you for the initial sample that we send you. If you need a refill, you should talk to your doctor about getting more.

(The following text was temporarily added 8/2021 when trace levels of nitrosamines were discovered within manufacturing process) There's something else we need to tell you about varenicline. Recently, quality control measures have discovered some impurities in the manufacturing process. Some of these impurities are known to be harmful if used over long periods, like years. We would be giving you varenicline over short duration, and we think this is safe, certainly safer than smoking. The FDA issued a voluntary warehouse recall to [the manufacturer], who makes varenicline, and new production has been halted for the moment. The FDA has not formally issued a recommendation for a patient level recall at this time. We therefore cannot get new supply into our pharmacy. The only supply we can offer is what we have in stock, and that is the varenicline with these known impurities. It is your choice how to proceed. (This text was removed once generic formulation was procured)

The most important thing to tell you now is that you can use the varenicline if you like, but you are not required to use it. We think it will help you, and we suggest you give a try for a least a few days, but it's really up to you if you decide to use it or not. If you decide to use the varenicline, we will send you a bottle of 56 pills, plus some very simple information about dosing instructions.

We'll also send you a letter that you can take to your physician if you want to get more. Your doctor can help you with a renewal prescription, and many insurance companies, including Medicaid, will cover the cost. In addition to a package of these materials, you will receive follow up surveys via email or text message according to the schedule in the consent form, starting two weeks from now.

Written: see 2pg brochure inserted into baseline mailing (following)

Free Sample of Medication
GIVE IT A TRY!

STARS
Smoking Treatment And Remote Sampling

Want to try varenicline? It's up to you!

We are offering you a chance to use varenicline (sometimes called Chantix). Varenicline is a quit smoking medication. Research has shown that varenicline is the best single medication for quitting smoking. It is even helpful for smokers who are not yet ready to quit. Varenicline does not contain nicotine, but it will help to control your cravings and desire to smoke. The vast majority of studies show that varenicline is safe to use. We have already determined that you are able to use this medication; you do not need a prescription from your doctor. **You are not required to take varenicline as part of this study. It is completely up to you if and how you take this medication. If you do take varenicline, follow the instructions below.**

Dosage and Instructions

Each pill provided to you is 0.5mg. If you choose to try varenicline, start with taking one pill daily for 3 days. After the third day, take two pills each day, one in the morning and one in the evening. Several studies show that this 1mg daily dose helps smokers quit, and results in fewer side effects. After a week of starting varenicline, you may want to increase to a stronger dose. If so, you can take up to two pills in the morning and two more pills in the evening (total of four pills/2mg daily). This 2mg dosage is even more effective but might result in more side effects: nausea and insomnia (see below). You shouldn't take more than 4 pills (2mg) daily. If you ever take a day off, or miss a day of medication, and want to re-start, just start this process over again. See the diagram below for more help.

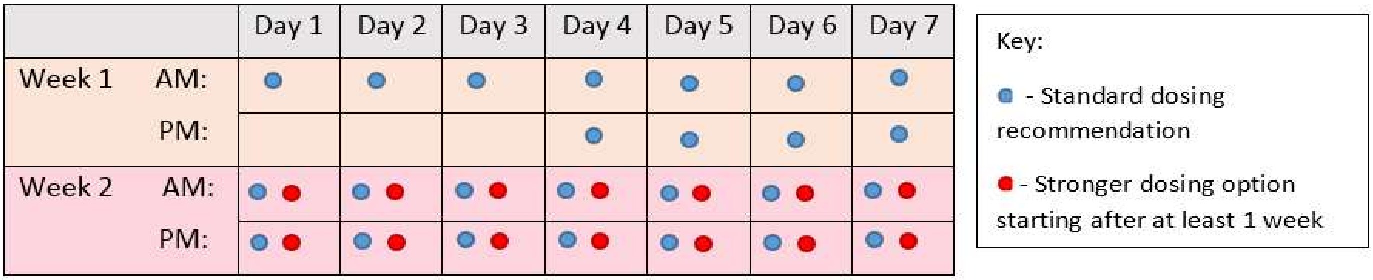

If you stop taking medication and wish to restart, start again on week 1, day 1.

Potential side effects: The two most common side effects of varenicline are nausea and insomnia (trouble falling asleep). To reduce nausea, take varenicline with a meal. To reduce insomnia, do not take it right before bedtime. If you do experience side effects, most symptoms go away after a few days of use.

If you want more varenicline: We hope this starter kit helps you. After using it, we hope that you continue to use it for as long as necessary. Talk to your doctor about getting more. Feel free to use the enclosed letter as a way of introduction with your physician. Most insurance programs, including Medicaid, will cover the costs of cessation medications, including varenicline.

© 2026 Carpenter MJ et al. *JAMA Network Open*.

Free Sample of Medication
More Information to Consider

STARS
Smoking Treatment And Remote Sampling

You have been given a free sample of medication to try:

- We hope this free sample of medication might get you thinking about quitting smoking. If you're ready to quit, that's great. If you're not ready to quit, that's ok too; you can use these and see if they help.
- You've already been screened to use them. We, and our study doctor, think they are safe to use.
- Even if you're not sure if you want to quit right now, try to use the medication(s), at least for a few days. Try them out and see what you think.
- See the other insert sheet that gives more information about the medication(s) you are receiving, including suggestions for how to use them.

Want to Keep it Going?

- When you're done with this free sample, consider using more.
- If you're in the group that receives patches and lozenges, these are available over-the-counter (you don't need a prescription) at all drug and grocery stores.
- If you're in the group that receives varenicline, you have also received a letter that you can take to your physician, who can prescribe more.
- For both groups, you might also consider calling the South Carolina Quitline (brochure enclosed, number below). Some smokers who call the Quitline may be eligible for free medications.
- For continued smoking cessation counseling and medication support, feel free to call the Medical University of South Carolina Tobacco Treatment Program: 843.xxx.xxxx. Mention the STARS study and ask for an appointment on Dr. XXXX's schedule. This is a telehealth treatment program available to people who smoke statewide. Insurance and payment options may apply.

Questions? Call the STARS study team: 1-800-xxx-xxxx
For additional cessation advice, you can always call the **Quitline:**
1-800 QUIT NOW (1-800- **xxx-xxxx**)

© 2026 Carpenter MJ et al. *JAMA Network Open*.

NRT messaging, both oral and written/mailed

Oral: *Baseline Phone Call / Group Assignment*
You have been assigned to the group that will receive the samples of nicotine replacement therapy, which is often just called NRT. There are many NRT options, but the two that we use are nicotine patch and nicotine lozenge. NRT provides nicotine that your body needs without all the harmful ingredients of a cigarette. NRT reduces cravings for cigarettes and other tobacco products. Studies consistently show that smokers who use NRT have a greater chance of quitting smoking, and that these effects are strongest when using the patch and lozenge together. The patch provides a steady dose of nicotine throughout the day, and lozenge can be used when needed when you have sudden increases in craving. Even if you're not ready to quit now, NRT can help smokers reduce how much they smoke. In fact, most guidelines and medical organizations consider combination patch and lozenge to be the among the best options for quitting smoking. There are three important things you need to know about patches and lozenges.

First, these products are available over the counter, which means you can get them almost anywhere like a grocery store or pharmacy chain, without a doctor's prescription.

Second, NRT is generally safe for use. There are some side effects that are possible, and the most common of these are nausea and insomnia. But the overall safety of NRT is well-established.

Third, smokers in our study will be given these patches and lozenges free of charge; there will be no cost to you for the initial sample that we send.

If you're willing, we'll send you everything you need via mail, and you'll receive it in a few days. In that mailing we will send complete instructions on use. The most important thing to tell you now is that you can use the NRT if you like, but you are not required to use it. We think it will help you, and we suggest you give a try for a least a few days, but it's really up to you if you decide to use it or not. Use of both patch and lozenge is best, but using one or the other is fine too.

In addition to a package of these materials, you will receive follow up surveys via email or text message according to the schedule in the consent form, starting two weeks from now.

Written: see 2pg brochure inserted into baseline mailing (following)

Free Sample of Medication
GIVE IT A TRY!

STARS
Smoking Treatment And Remote Sampling

Want to try patches or lozenges (or both)? It's up to you!

- We hope this free sample of medication might get you thinking about quitting smoking. If you're ready to quit, that's great. If you're not ready to quit, that's ok too.
- You've already been screened to use them. We, and our study doctor, think it's safe to use.
- Even if you're not sure if you want to quit right now, try to use the medications, at least for a few days. Try them out and see what you think.
- Using the nicotine patch and lozenge together at the same time is fine. This means you'll get more nicotine and be even less likely to want a cigarette. Studies show that combined use of patch and lozenge significantly increases the chances of quitting.
- When you're done with this free sample, consider buying more. These products are available over-the-counter (you don't need a prescription) at all drug and grocery stores. You'll save money in the long run – they're cheaper than cigarettes!

Suggested Use of Nicotine Patch

- Designed to give you a slow but steady dose of nicotine throughout the day.
- Put a patch on in the morning and take it off the next morning. It's that easy.
- You can sometimes have vivid dreams if you wear the patch overnight. If this happens, just take it off before you go to bed and put a new one on the next morning.
- Skin itchiness or redness is the most common complication, but this is usually very mild. You might need to rotate where you place the patch on your body.

Suggested Use of Nicotine Lozenge

- Pop a lozenge in your mouth in moments when you really want a cigarette. Even better – anticipate these triggers in advance and use it a few minutes earlier.
- Just suck on the lozenge (like a cough drop) until it dissolves. Occasionally move the lozenge to a different place in your mouth, to decrease the risk of mouth irritation.
- Do not bite or swallow the lozenge, as this may increase the risk of heartburn or indigestion.
- Works best if you don't eat or drink anything besides water 15 minutes prior to or while using.
- Throat irritation or burning is the most common complication, but this is usually very mild. Stick with it.

© 2026 Carpenter MJ et al. *JAMA Network Open*.

Free Sample of Medication
More Information to Conside

STARS
Smoking Treatment And Remote Sampling

You have been given a free sample of medication to try:

- We hope this free sample of medication might get you thinking about quitting smoking. If you're ready to quit, that's great. If you're not ready to quit, that's ok too; you can use these and see if they help.
- You've already been screened to use them. We, and our study doctor, think they are safe to use.
- Even if you're not sure if you want to quit right now, try to use the medication(s), at least for a few days. Try them out and see what you think.
- See the other insert sheet that gives more information about the medication(s) you are receiving, including suggestions for how to use them.

Want to Keep it Going?

- When you're done with this free sample, consider using more.
- If you're in the group that receives patches and lozenges, these are available over-the-counter (you don't need a prescription) at all drug and grocery stores.
- If you're in the group that receives varenicline, you have also received a letter that you can take to your physician, who can prescribe more.
- For both groups, you might also consider calling the South Carolina Quitline (brochure enclosed, number below). Some smokers who call the Quitline may be eligible for free medications.
- For continued smoking cessation counseling and medication support, feel free to call the Medical University of South Carolina Tobacco Treatment Program: 843.xxx.xxxx. Mention the STARS study and ask for an appointment on Dr. XXXX's schedule. This is a telehealth treatment program available to people who smoke statewide. Insurance and payment options may apply.

Questions? Call the STARS study team: 1-800-xxx-xxxx
For additional cessation advice, you can always call the **Quitline:**
1-800 QUIT NOW (1-800- **xxx-xxxx**)

© 2026 Carpenter MJ et al. *JAMA Network Open*.

eAppendix 2. Alternative Models, With and Without Adjustment
Models Adjusted for Baseline Imbalances, Per Protocol: Any Psychiatric Comorbidity

	Models adjusted for baseline imbalances, per protocol [any psychiatric comorbidity]		
	Model 1	Model 2 (no interaction)	
Outcome	Interaction (group by time)	Main Effects	OR (95% CI)[1]
	p-value		
Any 24hr QA (cumulative incidence)	0.67	Time (p<0.001)	
		Group (p=0.24)	
		varenicline vs. control	1.74 (0.90 to 3.38)
		varenicline vs. NRT	1.34 (0.70 to 2.58)
Floating 7-day Point Prevalence Abstinence (cumulative)	0.91	Time (p<0.001)	
		Group (p=0.01)	
		varenicline vs. control	2.7 (1.39 to 5.35)
		varenicline vs. NRT	1.56 (0.83 to 2.94)
7-day Point Prevalence Abstinence (PPA)	0.49	Time (p<0.001)	
		Group (p=0.02)	
		varenicline vs. control	2.16 (1.14 to 4.08)
		varenicline vs. NRT	2.03 (1.10 to 3.77)
CO-verified 7-day PPA	0.37	Time (p<0.001)	
		Group (p=0.12)	
		varenicline vs. control	2.31 (0.98 to 5.46)
		varenicline vs. NRT	1.63 (0.73 to 3.68)
Reduction of CPD by at least 50% from Baseline	0.006	NA	NA
Motivation to Quit[2]	0.05	Time (p<0.001)	
		Group (p<0.001)	
Confidence to Quit[2]	<0.001	NA	NA
Cessation Fatigue[2]	0.001	NA	NA
Cigarettes per Day[2]	<0.001	NA	NA

[1] Odds ratios and 95% confidence intervals shown for primary comparisons of varenicline vs. NRT and for varenicline vs. control groups
[2] Mechanism outcomes are shown in Supplement 5

© 2026 Carpenter MJ et al. *JAMA Network Open*.

Unadjusted Models

	Unadjusted Models		
	Model 1	Model 2 (no interaction)	
Outcome	Interaction (group by time)	Main Effects	OR (95% CI)[1]
	p-value		
Any 24hr QA (cumulative incidence)	0.67	Time (p<0.001)	
		Group (p=0.23)	
		varenicline vs. control	1.76 (0.91 to 3.40)
		varenicline vs. NRT	1.36 (0.71 to 2.60)
Floating 7-day Point Prevalence Abstinence (cumulative)	0.91	Time (p<0.001)	
		Group (p=0.01)	
		varenicline vs. control	2.75 (1.41 to 5.35)
		varenicline vs. NRT	1.57 (0.83 to 2.95)
7-day Point Prevalence Abstinence (PPA)	0.49	Time (p<0.001)	
		Group (p=0.02)	
		varenicline vs. control	2.15 (1.14 to 4.05)
		varenicline vs. NRT	2.02 (1.09 to 3.75)
CO-verified 7-day PPA	0.37	Time (p<0.001)	
		Group (p=0.12)	
		varenicline vs. control	2.31 (0.98 to 5.43)
		varenicline vs. NRT	1.64 (0.73 to 3.68)
Reduction of CPD by at least 50% from Baseline	0.006	NA	NA
Motivation to Quit[2]	0.05	Time (p<0.001)	
		Group (p<0.001)	
Confidence to Quit[2]	<0.001	NA	NA
Cessation Fatigue[2]	0.001	NA	NA
Cigarettes per Day[2]	<0.001	NA	NA

[1] Odds ratios and 95% confidence intervals shown for primary comparisons of varenicline vs. NRT and for varenicline vs. control groups
[2] Mechanism outcomes are shown in Supplement 5

© 2026 Carpenter MJ et al. *JAMA Network Open*.

Models Adjusted for Baseline Motivation to Quit (stratification variable)

	Models adjusted for stratification (baseline Motivation to Quit) variable only		
	Model 1	Model 2 (no interaction)	
Outcome	Interaction (group by time)	Main Effects	OR (95% CI)[1]
	p-value		
Any 24hr QA (cumulative incidence)	0.59	Time (p<0.001)	
		Group (p=0.33)	
		varenicline vs. control	1.64 (0.85 to 3.15)
		varenicline vs. NRT	1.23 (0.65 to 2.34)
Floating 7-day Point Prevalence Abstinence (cumulative)	0.91	Time (p<0.001)	
		Group (p=0.02)	
		varenicline vs. control	2.73 (1.38 to 5.43)
		varenicline vs. NRT	1.46 (0.77 to 2.78)
7-day Point Prevalence Abstinence (PPA)	0.49	Time (p<0.001)	
		Group (p=0.02)	
		varenicline vs. control	2.21 (1.15 to 4.26)
		varenicline vs. NRT	1.92 (1.02 to 3.60)
CO-verified 7-day PPA	0.35	Time (p<0.001)	
		Group (p=0.11)	
		varenicline vs. control	2.46 (1.01 to 5.95)
		varenicline vs. NRT	1.63 (0.72 to 3.72)
Reduction of CPD by at least 50% from Baseline	0.006	NA	NA
Motivation to Quit[2]	0.05	Time (p<0.001)	
		Group (p<0.001)	
Confidence to Quit[2]	<0.001	NA	NA
Cessation Fatigue[2]	0.002	NA	NA
Cigarettes per Day[2]	<0.001	NA	NA

[1] Odds ratios and 95% confidence intervals shown for primary comparisons of varenicline vs. NRT and for varenicline vs. control groups

[2] Mechanism outcomes are shown in Supplement 5

© 2026 Carpenter MJ et al. *JAMA Network Open*.

eAppendix 3. CO Adherence With Self-Reported Abstinence, per Group, per Time Point

	Control (N= 161)	Varenicline (N=318)	NRT (N=172)
**Week 2**	140 survey respondents<br>• 50 iCOs completed<br>• 35.7% of Week 2 respondents<br>• 30.9% of total sample<br>• 1 instance of self-reported abstinence<br>• 1 submitted iCO<br>• 1 CO-confirmed abstinence	287 survey respondents<br>• 141 iCOs completed<br>• 49.1% of Week 2 respondents<br>• 44.3% of total sample<br>• 2 instances of self-reported abstinence<br>• 2 submitted iCO<br>• 1 CO-confirmed abstinence	145 survey respondents<br>• 64 iCOs completed<br>• 44.1% of Week 2 respondents<br>• 37.2 of total sample<br>• 3 instances of self-reported abstinence<br>• 1 submitted iCO<br>• 1 CO-confirmed abstinence
**Week 4**	135 survey respondents<br>• 56 iCOs completed<br>• 41.4% of Week 4 respondents<br>• 34.6% of total sample<br>• 6 instances of self-reported abstinence<br>• 4 submitted iCO<br>• 3 CO-confirmed abstinence	281 survey respondents<br>• 131 iCOs completed<br>• 46.6% of Week 4 respondents<br>• 41.2% of total sample<br>• 17 instances of self-reported abstinence<br>• 10 submitted iCO<br>• 9 CO-confirmed abstinence	148 survey respondents<br>• 53 iCOs completed<br>• 35.8% of Week 4 respondents<br>• 30.8% of total sample<br>• 7 instances of self-reported abstinence<br>• 5 submitted iCO<br>• 5 CO-confirmed abstinence
**Week 8**	125 survey respondents<br>• 48 iCOs completed<br>• 38.4% of Week 8 respondents<br>• 29.6% of total sample<br>• 9 instances of self-reported abstinence<br>• 6 submitted iCO<br>• 4 CO-confirmed abstinence	259 survey respondents<br>• 107 iCOs completed<br>• 41.3% of Week 8 respondents<br>• 33.6% of total sample<br>• 40 instances of self-reported abstinence<br>• 24 submitted iCO<br>• 23 CO-confirmed abstinence	129 survey respondents<br>• 48 iCOs completed<br>• 37.2% of Week 8 respondents<br>• 27.9% of total sample<br>• 12 instances of self-reported abstinence<br>• 6 submitted iCO<br>• 5 CO-confirmed abstinence
**Week 12**	117 survey respondents<br>• 43 iCOs completed<br>• 36.8% of Week 12 respondents<br>• 26.5% of total sample<br>• 10 instances of self-reported abstinence	251 survey respondents<br>• 92 iCOs completed<br>• 36.7% of Week 12 respondents<br>• 28.9% of total sample<br>• 44 instances of self-reported abstinence	130 survey respondents<br>• 39 iCOs completed<br>• 30.0% of Week 12 respondents<br>• 22.7% of total sample<br>• 17 instances of self-reported abstinence

© 2026 Carpenter MJ et al. *JAMA Network Open*.

	• 6 submitted iCO<br>• 4 CO-confirmed abstinence	• 26 submitted iCO<br>• 25 CO-confirmed abstinence	• 9 submitted iCO<br>• 8 CO-confirmed abstinence
**Week 24**	119 survey respondents<br>• 33 iCOs completed<br>• 27.7% of Week 24 respondents<br>• 20.4% of total sample<br>• 16 instances of self-reported abstinence<br>• 7 submitted iCO<br>• 7 CO-confirmed abstinence	233 survey respondents<br>• 68 iCOs completed<br>• 29.2% of Week 24 respondents<br>• 21.4% of total sample<br>• 53 instances of self-reported abstinence<br>• 19 submitted iCO<br>• 18 CO-confirmed abstinence	121 survey respondents<br>• 36 iCOs completed<br>• 29.8% of Week 24 respondents<br>• 20.9% of total sample<br>• 14 instances of self-reported abstinence<br>• 5 submitted iCO<br>• 4 CO-confirmed abstinence
**All Weeks**	42 instances of self-reported abstinence<br>Of which 24 (57.1%) submitted CO samples<br>Of which 19 (45.2%) were CO confirmed	156 instances of self-reported abstinence<br>Of which 81 (51.9% submitted CO samples<br>Of which 76 (48.7%) were CO confirmed	53 instances of self-reported abstinence<br>Of which 26 (49.1%) submitted CO samples<br>Of which 23 (43.3%) were CO confirmed
**All Weeks, All Groups**	251 instances of self-reported abstinence<br>Of which 131 (52.2%) submitted CO samples<br>Of which 118 (47.0%) were CO confirmed<br>13 (5.2%) instances of discordance (self-report vs. CO)		

© 2026 Carpenter MJ et al. *JAMA Network Open*.

eAppendix 4. Treatment Mechanisms

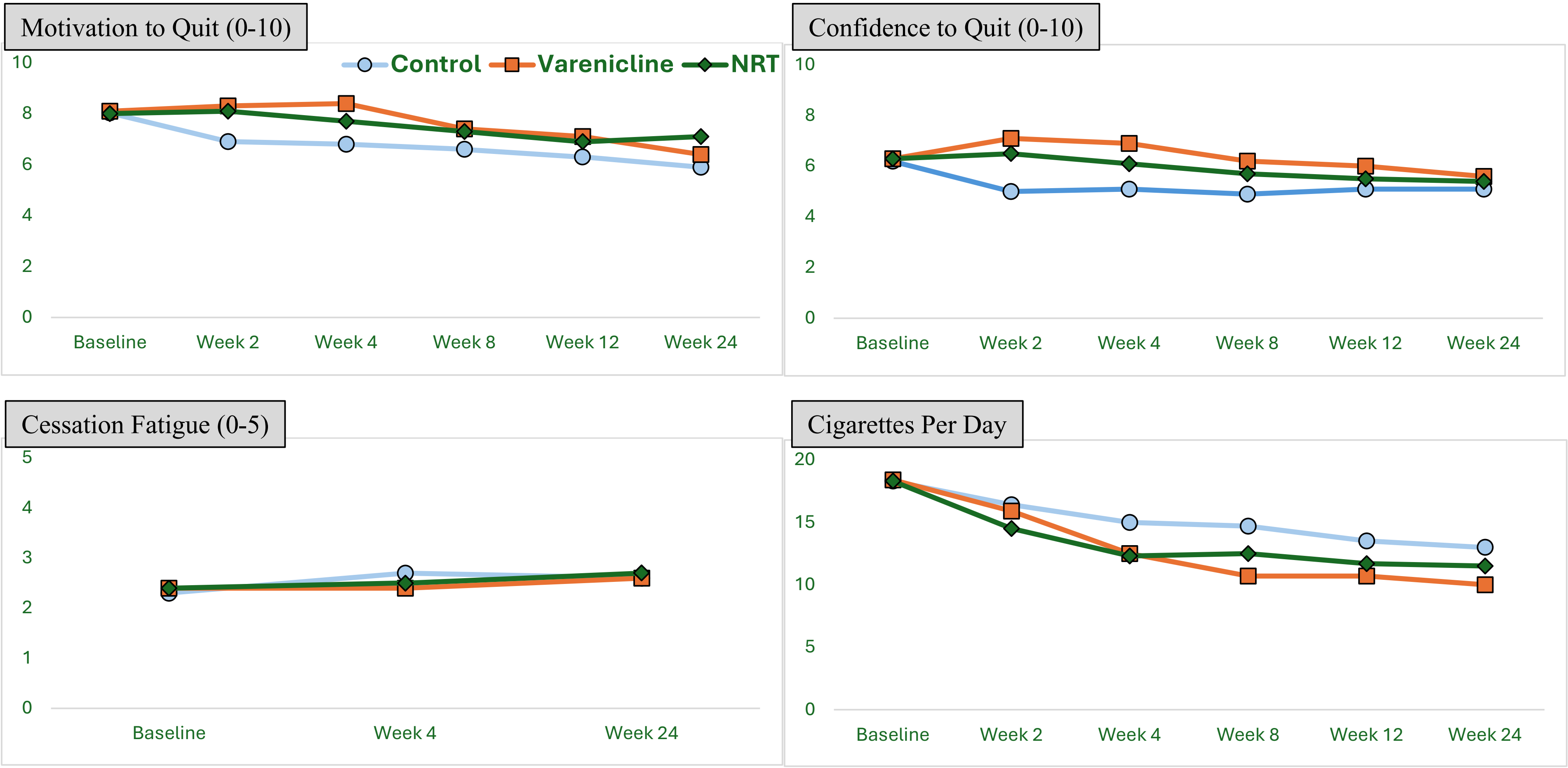

Motivation to quit: p= 0.05 for group by time interaction
Cessation fatigue: p=0.001 for group by time interaction
All others: p<0.001 for group by time interaction

© 2026 Carpenter MJ et al. *JAMA Network Open*.

eAppendix 5. Analyses by Motivation to Quit

Self-Reported Quit Attempts, Floating Abstinence and Smoking Reduction, by baseline Motivation to Quit (MTQ)

	**Low MTQ[1] (N=145)**					**High MTQ[1] (N=506)**			
	**All (N=145)**	**Control (N=37)**	**Varenicline (N=71)**	**NRT (N=37)**		**All (N=506)**	**Control (N=124)**	**Varenicline (N=247)**	**NRT (N=135)**
	**N (%)**	**N (%)**	**N (%)**	**N (%)**		**N (%)**	**N (%)**	**N (%)**	**N (%)**
7-day Point Prevalence Abstinence at Week 2	0 (0)	0 (0)	0 (0)	0 (0)		6 (1)	1 (1)	2 (1)	3 (2)
7-day Point Prevalence Abstinence at Week 4	3 (2)	0 (0)	2 (3)	1 (3)		27 (5)	6 (5)	15 (6)	6 (4)
7-day Point Prevalence Abstinence at Week 8	4 (3)	0 (0)	3 (4)	1 (3)		57 (11)	9 (7)	37 (15)	11 (8)
7-day Point Prevalence Abstinence at Week 12	6 (4)	0 (0)	4 (6)	2 (5)		65 (13)	10 (8)	40 (16)	15 (11)
7-day Point Prevalence Abstinence at Week 24	8 (6)	1 (3)	5 (7)	2 (4)		75 (15)	15 (12)	48 (19)	12 (9)
Reduction of CPD by at least 50% at Week 2	8 (6)	0 (0)	4 (6)	4 (11)		56 (11)	8 (6)	27 (11)	21 (16)
Reduction of CPD by at least 50% at Week 4	22 (15)	2 (5)	13 (18)	7 (19)		135 (27)	16 (13)	79 (32)	40 (30)
Reduction of CPD by at least 50% at Week 8	27 (19)	2 (5)	17 (24)	8 (22)		145 (29)	21 (17)	94 (38)	30 (22)
Reduction of CPD by at least 50% at Week 12	31 (21)	5 (14)	16 (23)	10 (27)		162 (32)	31 (25)	94 (38)	37 (27)
Reduction of CPD by at least 50% at Week 24	32 (22)	4 (11)	18 (25)	10 (27)		148 (29)	27 (22)	88 (36)	33 (24)
Floating 7-day Point Prevalence Abstinence through Week 2	5 (3)	0 (0)	4 (6)	1 (3)		33 (7)	6 (5)	18 (7)	9 (7)
Floating 7-day Point Prevalence Abstinence through Week 4	8 (6)	0 (0)	5 (7)	3 (8)		60 (12)	13 (10)	32 (13)	15 (11)
Floating 7-day Point Prevalence Abstinence through Week 8	13 (9)	0 (0)	8 (11)	5 (14)		96 (19)	17 (14)	56 (23)	23 (17)
Floating 7-day Point Prevalence Abstinence through Week 12	13 (9)	0 (0)	8 (11)	5 (14)		118 (24)	20 (16)	71 (29)	27 (20)
Floating 7-day Point Prevalence Abstinence through Week 24	18 (12)	2 (5)	10 (14)	6 (16)		166 (33)	31 (25)	98 (40)	37 (27)
Any 24-hour QA at Week 2	6 (4)	0 (0)	4 (6)	2 (5)		55 (11)	11 (9)	29 (12)	15 (11)
Any 24-hour QA through Week 4 (cumulative)	16 (11)	0 (0)	9 (13)	7 (19)		122 (24)	30 (24)	58 (23)	34 (25)
Any 24-hour QA through Week 8 (cumulative)	26 (18)	4 (11)	13 (18)	9 (24)		207 (41)	47 (38)	112 (45)	48 (36)
Any 24-hour QA through Week 12 (cumulative)	32 (22)	5 (14)	17 (24)	10 (27)		251 (50)	57 (46)	135 (55)	59 (44)
Any 24-hour QA through Week 24 (cumulative)	42 (29)	9 (24)	21 (30)	12 (32)		293 (58)	68 (55)	157 (64)	68 (50)

[1] Low: 0-6; High: 7-10 on 0-10 VAS scale

All percents are based on full sample denominators, where missing data points were treated as not having the outcome of interest at that timepoint

CO-verified abstinence not shown due to low rates of abstinence within groups

© 2026 Carpenter MJ et al. *JAMA Network Open*.

Mechanisms of sampling: Changes in Motivation and Confidence to Quit (possible range 0-10), Cessation Fatigue (range 1-5), by baseline Motivation to Quit (MTQ)

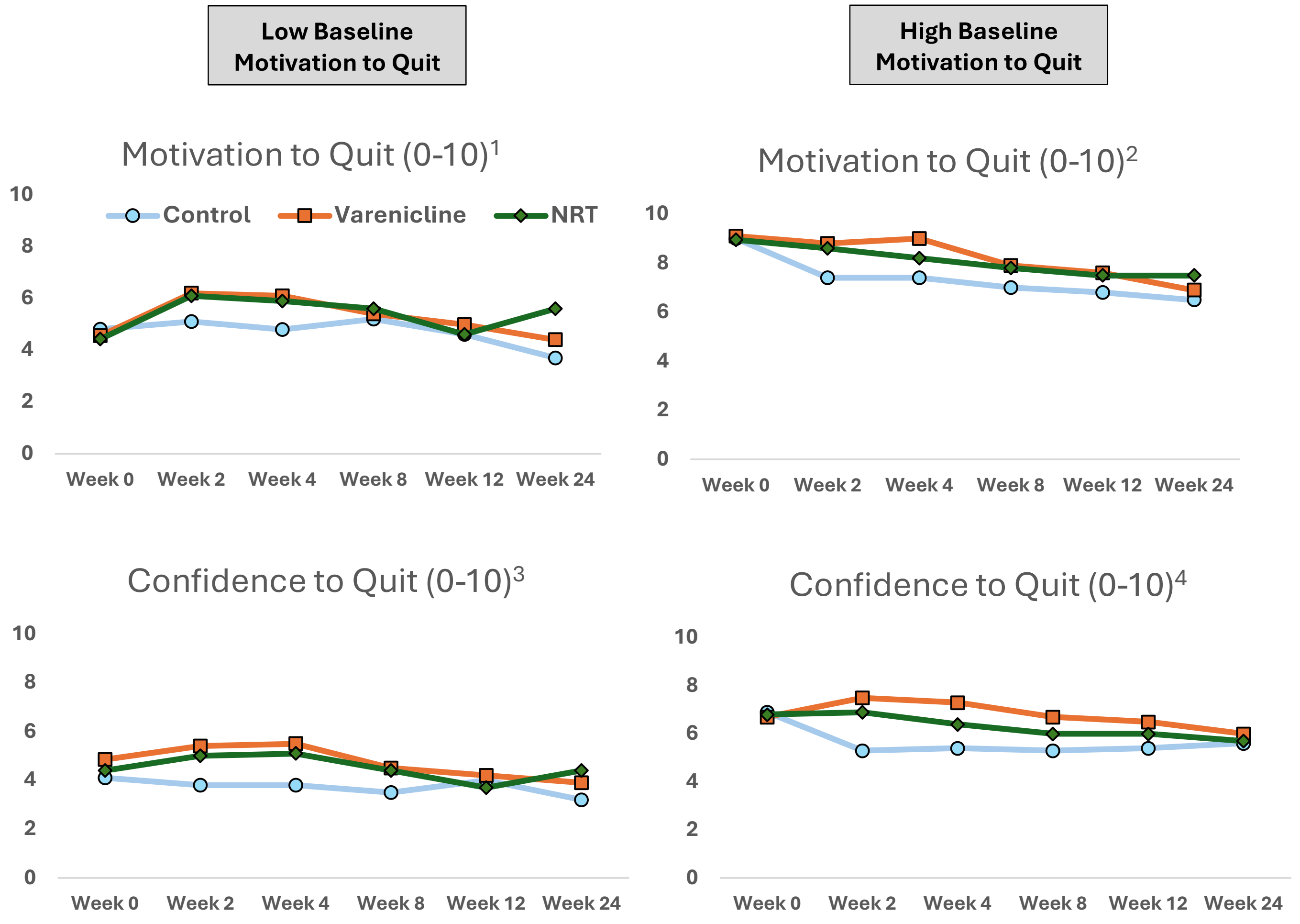

© 2026 Carpenter MJ et al. *JAMA Network Open*.

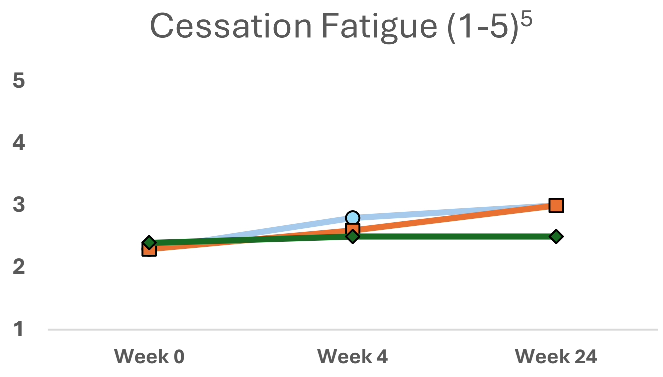

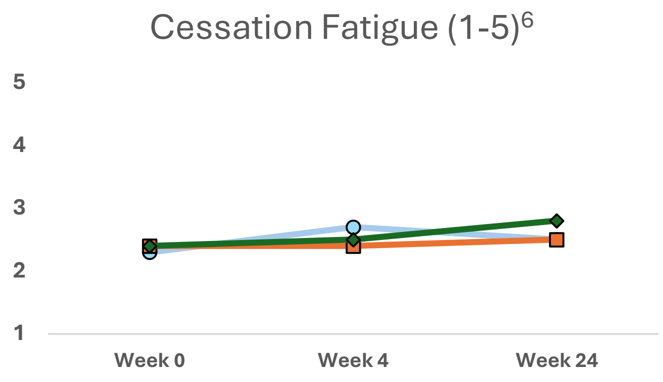

[1] Motivation to Quit in Low Baseline MTQ group interaction term (time by group) significance p=0.20
[2] Motivation to Quit in High Baseline MTQ group interaction term (time by group) significance p=0.17
[3] Confidence to Quit in Low Baseline MTQ group interaction term (time by group) significance p=0.84
[4] Confidence to Quit in High Baseline MTQ group interaction term (time by group) significance p<0.001
[5] Cessation Fatigue in Low Baseline MTQ group interaction term (time by group) significance p=0.09
[6] Cessation Fatigue in High Baseline MTQ group interaction term (time by group) significance p<0.001

© 2026 Carpenter MJ et al. *JAMA Network Open*.

Use of Either Varenicline or NRT (specifically, patch and/or lozenge): Any Use and Regular Use (≥4 days per week), by baseline Motivation to Quit (MTQ)

	Low MTQ (N=145)					High MTQ (N=506)			
	**All (N=145)**	**Control (N=37)**	**Varenicline (N=71)**	**NRT (N=37)**		**All (N=506)**	**Control (N=124)**	**Varenicline (N=247)**	**NRT (N=135)**
	**N (%)**	**N (%)**	**N (%)**	**N (%)**		**N (%)**	**N (%)**	**N (%)**	**N (%)**
Use of medication (either VRN and/or NRT - specific to Patch and Lozenge) at Week 2	64 (54)	1 (3)	42 (71)	21 (70)		260 (58)	8 (7)	175 (77)	77 (67)
Use of either medication at Week 4	61 (52)	0 (0)	40 (67)	21 (70)		276 (64)	10 (10)	178 (82)	88 (76)
Use of either medication at Week 8	45 (41)	3 (12)	23 (43)	19 (66)		160 (40)	7 (7)	94 (46)	59 (59)
Use of either medication at Week 12	26 (24)	2 (8)	13 (24)	11 (39)		123 (31)	6 (6)	66 (34)	51 (50)
Use of either medication at Week 24	32 (33)	2 (10)	15 (31)	15 (54)		108 (30)	16 (18)	48 (27)	44 (47)
Regular Use[1] of sampling medication (either VRN and/or NRT - patch and/or lozenge) at Week 2	36 (56)	0 (0)	28 (67)	8 (38)		193 (74)	3 (38)	133 (76)	57 (74)
Regular Use of sampling medication at Week 4	40 (67)	0 (0)	31 (79)	9 (43)		205 (75)	4 (40)	141 (80)	60 (68)
Regular Use of sampling medication at Week 8	23 (51)	3 (100)	14 (61)	6 (32)		94 (59)	4 (57)	58 (62)	32 (54)
Regular Use of sampling medication at Week 12	14 (54)	1 (50)	10 (77)	3 (27)		69 (56)	4 (67)	38 (58)	27 (53)
Regular Use of sampling medication at Week 24	11 (34)	1 (50)	6 (40)	4 (27)		40 (37)	6 (38)	19 (40)	15 (34)

[1] Regular use defined as using 4+ days per week, asked only among medication users. (Example: within highlighted cell, 15 is 34% of 44 users at Week 24)

© 2026 Carpenter MJ et al. *JAMA Network Open*.

eAppendix 6. Adverse Events

	# Adverse Events (AEs)	# of Related[1] AEs	# of Participants with Related AEs	Descriptor of Top Three Related AEs	# Serious[2] AEs
Control Group	207	7[3]	1	n/a[4]	0
NRT Group	345	10	7	1) Vivid dreams (4 ppts);<br>2) Headache (3 ppts);<br>3) Dizziness (2 ppts)	0
Varenicline Group	859	619	191	1) Vivid dreams (81 ppts);<br>2) Insomnia (75 ppts);<br>3) Nausea (64 ppts)	0
**Total**	1411	636	199		0

[1] Possibly, Probably, or Definitely Related
[2] IRB-Defined Serious Adverse Events: Related adverse events involving hospitalization, persistent or significant incapacity, or resulting in death
[3] Adverse events in the control group were deemed related if they were using varenicline and/or NRT, on their accord
[4] One person reported seven separate adverse events, each once only

© 2026 Carpenter MJ et al. *JAMA Network Open*.
