# Supplementary material for: Medication Samples and Smoking Cessation Among Adults: A Randomized Clinical Trial
Source: JAMA Netw Open. 2026 May 8;9(5):e2611418. doi: 10.1001/jamanetworkopen.2026.11418 (PMC13156786; doi:10.1001/jamanetworkopen.2026.11418)
Supplement: Supplement 3. — Data Sharing Statement [file jamanetwopen-e2611418-s003.pdf]

Data Sharing Statement

Carpenter. Medication Samples and Smoking Cessation Among Adults Who Smoke. *JAMA Netw Open*. Published May 08, 2026. doi:10.1001/jamanetworkopen.2026.11418

Data
Additional Information: NCT 04525755
Data available: Yes
Data types: Deidentified participant data
How to access data: Deidentified participant data can be requested from the corresponding author (carpente@musc.edu
When available: With publication

Supporting Documents
Document types: None

Additional Information
Who can access the data: Data will be available for researchers whose proposed use of the data has been approved.
Types of analyses: For any specified purpose.
Mechanisms of data availability: Data will be made available without investigator support.
